# Supplementary material for: PPARα Inhibition Overcomes Tumor-Derived Exosomal Lipid-Induced Dendritic Cell Dysfunction
Source: Cell Rep. Author manuscript; Available in PMC 2020 Dec 29. (PMC7771208; doi:10.1016/j.celrep.2020.108278)
Supplement: 1 [file NIHMS1639624-supplement-1.pdf]

Cell Reports, Volume *33*

Supplemental Information

PPARα Inhibition Overcomes Tumor-Derived Exosomal Lipid-Induced Dendritic Cell Dysfunction

Xiaozhe Yin, Wenfeng Zeng, Bowen Wu, Luoyang Wang, Zihao Wang, Hongjian Tian, Luyao Wang, Yunhan Jiang, Ryan Clay, Xiuli Wei, Yan Qin, Fayun Zhang, Chunling Zhang, Lingtao Jin, and Wei Liang

Figure S1

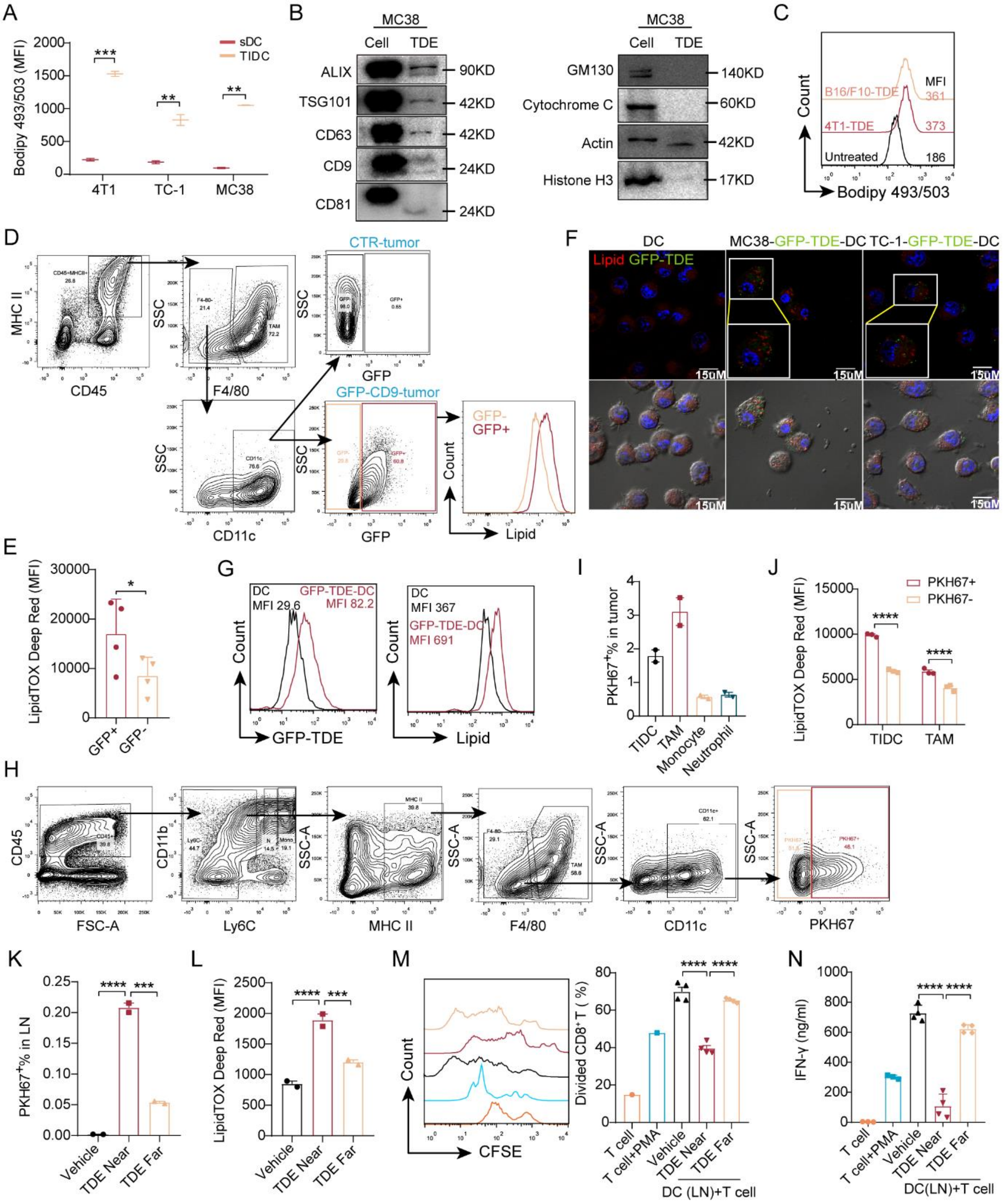

A
Bodipy 493/503 (MFI)
sDC
TIDC
4T1
TC-1
MC38
B
MC38
Cell
TDE
ALIX
TSG101
CD63
CD9
CD81
90KD
42KD
42KD
24KD
24KD
GM130
Cytochrome C
Actin
Histone H3
140KD
60KD
42KD
17KD
C
Count
MFI
B16/F10-TDE 361
4T1-TDE 373
Untreated 186
Bodipy 493/503
D
MHC II
CD45
SSC
F4/80
CTR-tumor
GFP
GFP-CD9-tumor
CD11c
GFP-
GFP+
Count
Lipid
E
LipidTOX Deep Red (MFI)
GFP+
GFP-
F
DC
MC38-GFP-TDE-DC
TC-1-GFP-TDE-DC
Lipid GFP-TDE
15uM
G
DC
MFI 29.6
GFP-TDE-DC
MFI 82.2
GFP-TDE
DC
MFI 367
GFP-TDE-DC
MFI 691
Lipid
I
PKH67+% in tumor
TIDC
TAM
Monocyte
Neutrophil
J
LipidTOX Deep Red (MFI)
PKH67+
PKH67-
TIDC
TAM
H
CD45
FSC-A
CD11b
Ly6C
SSC-A
MHC II
F4/80
CD11c
PKH67
K
PKH67+% in LN
Vehicle
TDE Near
TDE Far
L
LipidTOX Deep Red (MFI)
M
Count
CFSE
Divided CD8+T (%)
T cell
T cell+PMA
DC (LN)+T cell
N
IFN-γ (ng/ml)
DC(LN)+T cell

Figure S1 (related to Figure 1). TDEs induce lipid laden DC

(A) Intracellular lipid level of $CD45^+MHC\ II^+CD11c^+\ F4/80^-$ DCs in spleen (sDC) and tumor (TIDC) from various tumor models was measured by Bodipy 493/503 staining (n=3 per group).

(B) Western blot reveals exosome markers in both cell lysate and TDE.

(C) Intracellular lipid levels in BMDCs treated with 4T1 or B16/F10 tumor cells derived exosomes (400 μg/ml) for 24 h. Representative of two independent experiments.

(D) Flow cytometry and gating strategy of tumor infiltrating DCs in GFP-CD9-tumor or CTR-tumor.

(E) Intracellular lipid levels in GFP^+ or GFP^- TAMs were analyzed by flow cytometry, related to Figure 1I-K. Each dot represents an individual mouse.

(F) Representative confocal images showing the intracellular lipid level of DCs treated with 100 μg/ml GFP-TDE for 8 h. Scale bar = 15 μm.

(G) GFP-TDE uptake and lipid level of DCs treated with 400 μg/ml GFP-TDE for 24 h.

(H) MC38 tumors (on day 10) were harvested 48 h after 1000 μg PKH67 labeled TDEs injection. Flow cytometry and gating strategy of tumor infiltrating myeloid cells.

(I and J) Relative proportions of tumor infiltrating myeloid cells as a percentage of total $PKH67^+$ cells (I). TIDC ($CD45^+Ly6C^-MHC\ II^+CD11c^+F4/80^-$), TAM ($CD45^+Ly6C^-MHC\ II^+F4/80^+$), Monocytes ($CD45^+CD11b^+Ly6C^{hi}$), Neutrophils ($CD45^+CD11b^+Ly6C^{mid}$). Lipid content in $PKH67^+$ or $PKH67^-$ TIDC and TAM were analyzed by flow cytometry (n=4) (J). Each dot represents an individual mouse.

(K-N) 1000 μg PKH67 labeled TDEs were injected into the footpad of wild type C57BL/6 mice and measured the phenotype change of DCs in popliteal lymph nodes (LN)(near) and inguinal lymph nodes (far) at 48 h post injection. Frequency of DC populations as a percentage of total $PKH67^+$ cells (K). Lipid content was analyzed by flow cytometry (L). The isolated DCs ($CD45^+Ly6C^-MHC\ II^+CD11c^+F4/80^-$) from lymph nodes were treated with 2 mg/ml OVA overnight, then co-cultured with OT I $CD8^+$ T cells for 3 days. Proliferation of $CD8^+$ T was analyzed by CFSE dilution (M) and the production of IFN-γ of $CD8^+$ T cells was analyzed by ELISA (N). T: undivided OT-I cells as a negative control. PMA: OT-I T cells primed by PMA and ionomycin as a qualification control. Each dot represents an individual mouse.

$^{**}P < 0.01$; $^{***}P < 0.001$; $^{****}P < 0.0001$. (A) and (J) were analyzed with two-way ANOVA. (E) was analyzed with 2-tailed t test. Other data were analyzed with one-way ANOVA. Error bars represent SEM. The error bars of (N) represents SD.

Figure S2

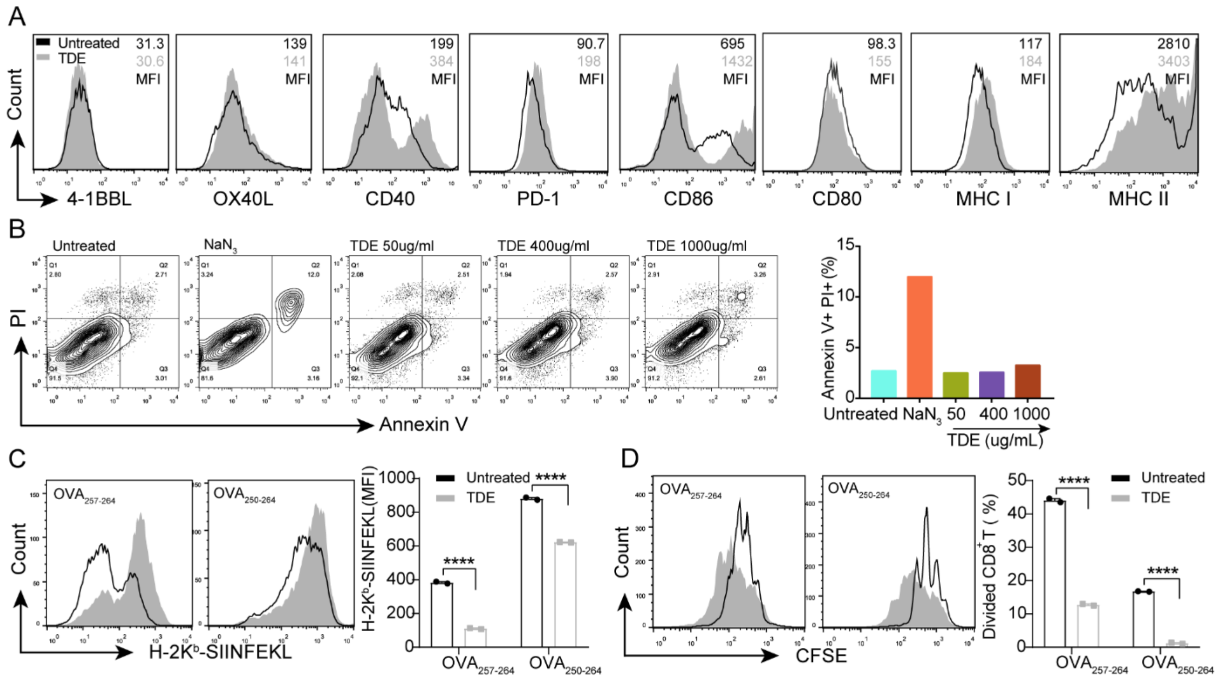

Figure S2 (related to Figure 2). TDEs interfere DC immune function

(A) BMDCs were treated with TDEs (400 μg/ml) for 24 h. The expression of surface markers for BMDCs maturation were analyzed. Representative of two independent experiments.

(B) Cytotoxicity analysis of TDEs in BMDCs with 24 h. Representative of two independent experiments.

(C) BMDCs were treated with 10 μg/ml $OVA_{257-264}$ or $OVA_{250-264}$ in the presence or absence of TDEs (200 μg/ml) for 24 h. Antigen presentation of BMDCs was assessed by staining with H-2Kb-SIINFEKL antibody. Representative of two independent experiments.

(D) BMDCs were treated with 10 μg/ml $OVA_{257-264}$ or $OVA_{250-264}$ in the presence or absence of TDEs (200 μg/ml) for 48 h, which were subsequently washed off the antigen and co-cultured with OT I $CD8^+$ T cells for 3 days. Proliferation of OT I $CD8^+$ T cells was analyzed by CFSE dilution. Representative of two independent experiments.

****$P < 0.0001$. Data were analyzed with two-way ANOVA. Error bars represent SEM.

Figure S3

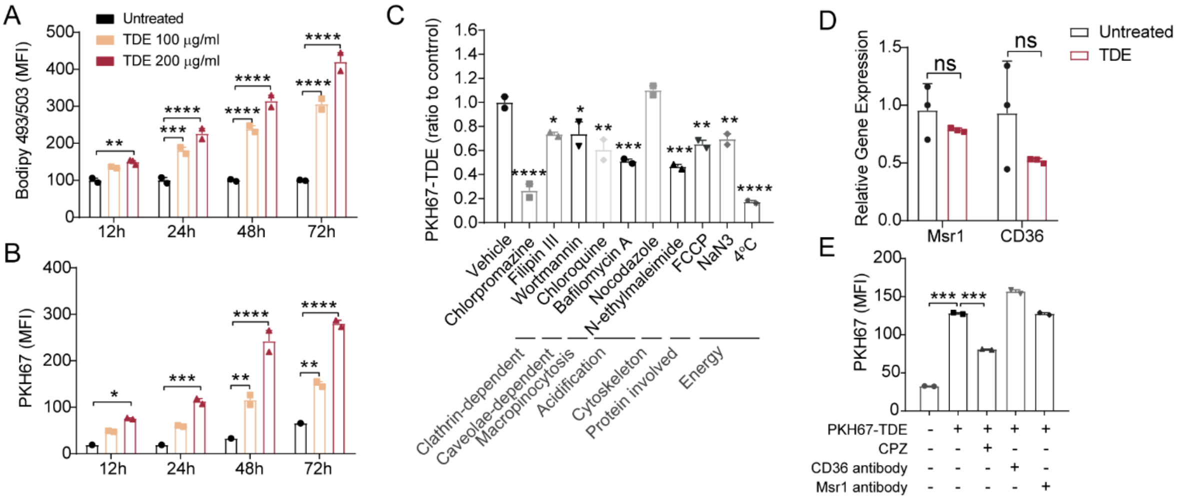

Figure S3 (related to Figure 3). The mechanism of TDEs uptake by DCs

(A and B) BMDCs were treated with 100 μg/ml or 200 μg/ml PKH67-labeled TDEs for different periods. 12, 24, 48 and 72 hours later, BMDCs were collected and the fluorescence intensity of Bodipy 493/503 (A) and PKH67 (B) were analyzed by flow cytometry respectively.

(C) Effects of endocytic inhibitors on internalization of PKH67-labeled TDEs. BMDCs were pre-treated with various inhibitors for different lengths of time (see Table 1), and then incubated with 100 μg/ml PKH67-labeled exosomes for 8 h. Internalized exosomes were detected by flow cytometry. Representative of two independent experiments.

(D) Expression of the indicated transcripts were assessed by RNA-Seq.

(E) Flow cytometry analyzed the effect of CD36 antibody or Msr1 antibody on internalization of PKH67 labeled TDEs. BMDCs were pre-treated with CD36 antibody or Msr1 antibody for 15 min and then incubated with 50 μg/ml PKH67 labeled TDEs for 8 h. CPZ, Chlorpromazine.

*$P < 0.05$; **$P < 0.01$; ***$P < 0.001$; ****$P < 0.0001$; ns, not significant. (C) and (E) were analyzed with one-way ANOVA. Other data were analyzed with two-way ANOVA. Error bars represent SEM.

Figure S4

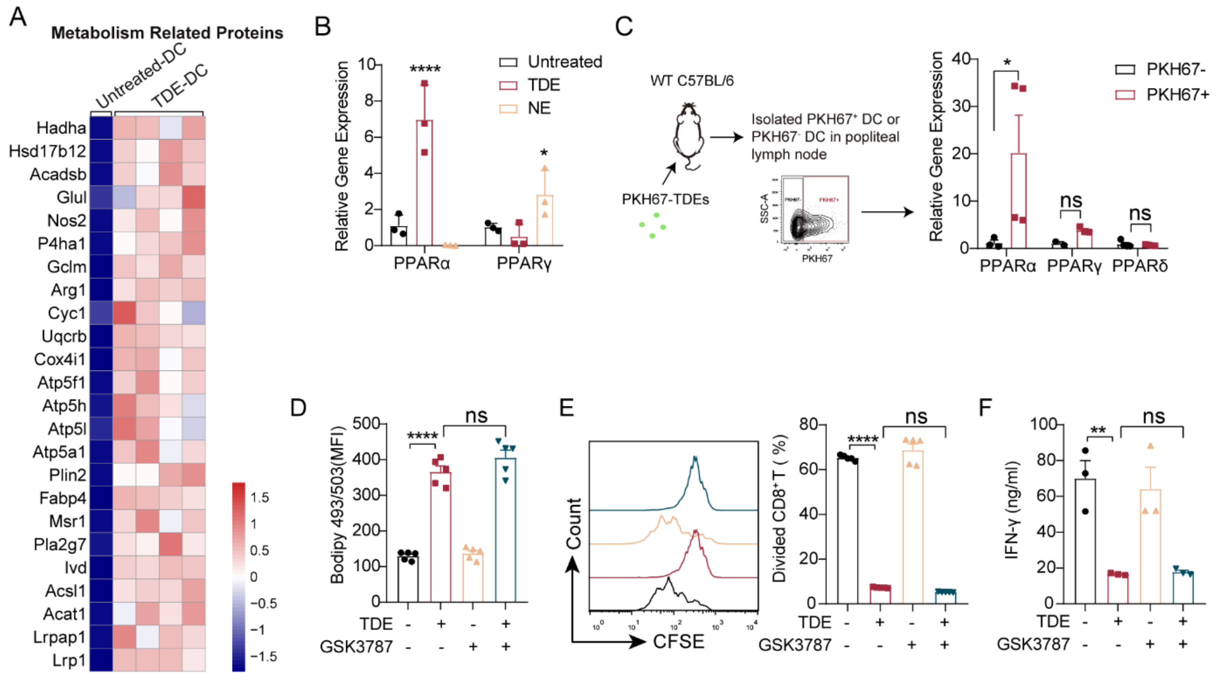

Figure S4 (related to Figure 4). Proteomics analysis of BMDC

(A) Upregulation of genes involved in the metabolism of BMDCs cultured with or without TDEs were analyzed by proteomics.

(B) Expression of the indicated transcripts were assessed by qRT-PCR.

(C) 1000 μg PKH67 labeled TDEs were injected into the footpad of wild type C57BL/6 mice and isolated PKH67+ or PKH67- DCs in popliteal lymph nodes (LN). Expression of the indicated transcripts were assessed by qRT-PCR.

(D) BMDCs were incubated with or without TDEs (400 μg/ml) in the presence or absence of GSK3787 (1 μM) for 48 h. Intracellular lipid content was assessed 24 h later via Bodipy 493/503 staining.

(E and F) BMDCs were treated as in (D) with the addition of 2 mg/ml OVA, which were subsequently washed off the antigen and co-cultured with OT I $CD8^+$ T cells for another 3 days. Proliferation of OT I $CD8^+$ T cells was analyzed by CFSE dilution (E). The production of IFN-γ by $CD8^+$ T cells was assessed by ELISA (F).

*$P < 0.05$; ***$P < 0.001$; ****$P < 0.0001$; ns, not significant. (B) and (C) were analyzed with two-way ANOVA. Other data were analyzed with one-way ANOVA. Error bars represent SEM. The error bars of (F) represents SD.

Figure S5

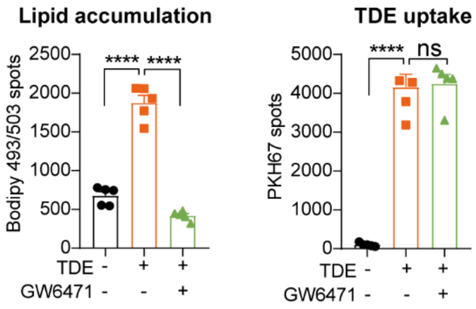

Figure S5 (related to Figure 5). Lipid accumulation and TDE uptake of BMDC

BMDCs were treated with or without TDEs (400 μg/ml) in the presence or absence of GW6471 (15 μM) for 8 h, the intracellular lipid content and TDE uptake were detected by Bodipy493/503 or PKH67, and recorded by Opera Phenix™ High Content Screening System. Each dot represents a biological repeat.

*****P* < 0.0001; ns, not significant. Data were analyzed with one-way ANOVA. Error bars represent SEM.

Figure S6

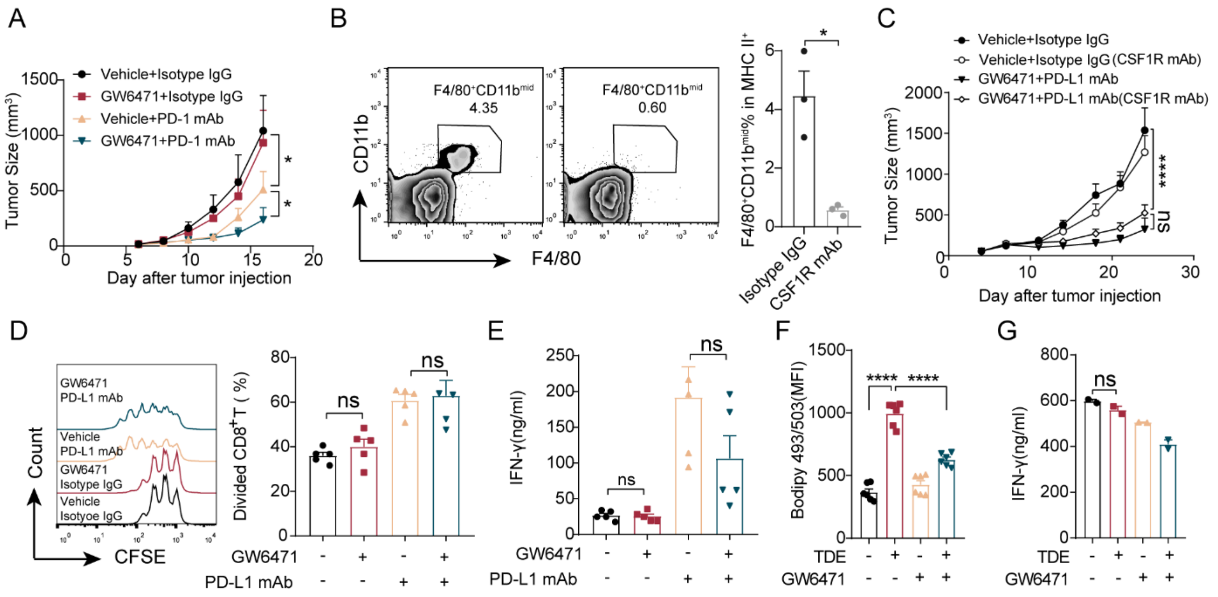

Figure S6 (related to Figure 1 and 6). Macrophages are dispensable for the combination therapy

(A) B16/F10 bearing C57BL/6 mice were treated with PD-1 mAb (200 μg/dose per mouse on day 3,6,9) and GW6471 (10 mg/kg, from day 6, once every other day). Tumor growth curve was shown (*n*=5 per group).

(B) MC38-OT I bearing C57BL/6 mice were treated with PD-L1 mAb (200 μg/dose per mouse on day 7,10,17) and GW6471 (10 mg/kg, from day 11, once every other day). CSF1R mAb 200 ug/mice was injected intraperitoneally on day 7, 10 and 17. 24 h after the third CSF1R mAb injection, $CD45^{+}$MHC $II^{+}CD11b^{mid}F4/80^{+}$ cells were measured by flow cytometry. Each dot represents an individual mouse.

(C) Tumor growth curve was shown (*n*=5 per group).

(D and E) The isolated TAM ($CD45^{+}$MHC $II^{+}F4/80^{+}$) were treated with 2 mg/ml OVA overnight, then co-cultured with OT I $CD8^{+}$ T cells for 3 days. Proliferation of $CD8^{+}$ T was analyzed by CFSE dilution (D) and the production of IFN-γ of $CD8^{+}$ T cells was analyzed by ELISA (E).

(F) BMDMs were differentiated by M-CSF for 7 days, and lipid level in BMDMs treated with TDEs (400 μg/ml) for 24 h in the presence or absence of GW6471.

(G) BMDMs were treated with 2 mg/ml OVA in the presence or absence of TDEs (400 μg/ml) for 48 h, which were subsequently washed off the antigen and co-cultured with OT I $CD8^{+}$ T cells for 3 days. The production of IFN-γ by $CD8^{+}$ T cells was analyzed by ELISA.

**P* < 0.05; *****P* < 0.0001; ns, not significant. Data were analyzed with 2-tailed *t* test. Error bars represent SEM. The error bar of (G) represents SD.

Table S1

Endocytosis inhibitor	Mechanism	Concentration and incubation time
chlorpromazine	interacts with clathrin	25 uM, 30 min
filipin III	binds to 3' β-hydroxysterol	8 uM, 30 min
wortmannin	blocks PI-3 kinase	100 nM, 60 min
chloroquine	increases endosomal pH	100 uM, 30 min
bafilomycin A	blocks v(H+)ATPase	100 nM, 60 min
nocodazole	depolymerizes microtubules	20 uM, 30 min
N-ethylmaleimide	alkylates sulfhydryl	2 mM, 30 min
FCCP	an uncoupling agent	10 uM, 15 min
NaN_3	depletes cellular ATP	10 mM, 15 min

Table S1 (related to Figure 3). The inhibitors used to block TDEs endocytosis.
