# Supplementary material for: PPARα Inhibition Overcomes Tumor-Derived Exosomal Lipid-Induced Dendritic Cell Dysfunction
Source: Cell Rep. Author manuscript; Available in PMC 2020 Dec 29. (PMC7771208; doi:10.1016/j.celrep.2020.108278)
Supplement: 4 [file NIHMS1639624-supplement-4.pdf]

Cell Reports

Article

PPARα Inhibition Overcomes Tumor-Derived Exosomal Lipid-Induced Dendritic Cell Dysfunction

Graphical Abstract

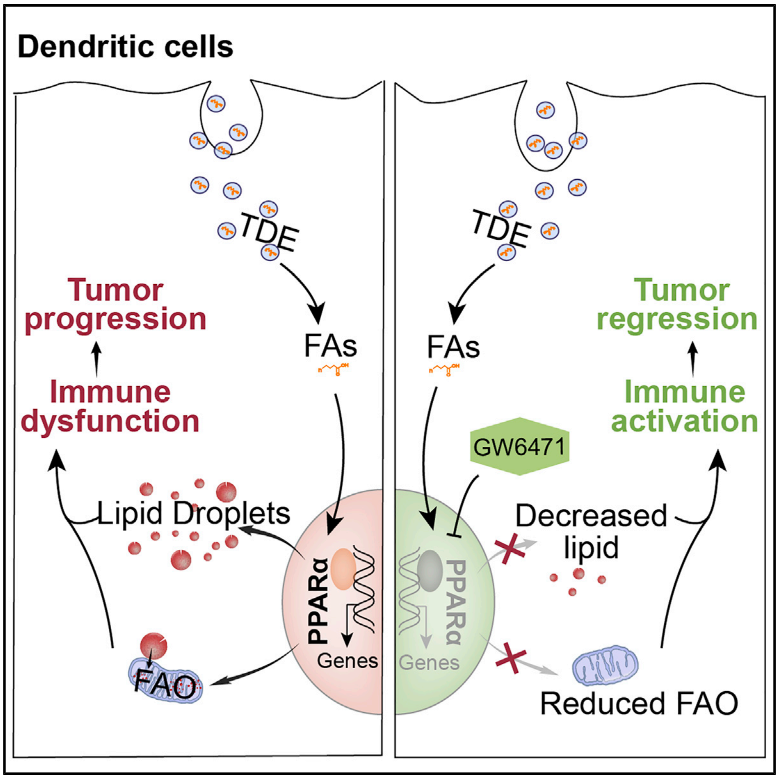

Highlights

- Tumor-derived exosomes (TDEs) drive DC dysfunction
- TDE-derived fatty acids activate PPARα to induce DC fatty acid oxidation
- PPARα blockade modulates lipid metabolism and function in DCs
- PPARα inhibition enhances the anti-tumor efficacy of immunotherapies

Authors

Xiaozhe Yin, Wenfeng Zeng, Bowen Wu, ..., Chunling Zhang, Lingtao Jin, Wei Liang

Correspondence

zengwenfeng@ibp.ac.cn (W.Z.), ljin1@ufl.edu (L.J.), weixx@sun5.ibp.ac.cn (W.L.)

In Brief

Yin et al. reveal that tumor-derived exosomes (TDEs), as fatty acid carriers, induce a metabolic shift toward oxidative phosphorylation, driving DC immune dysfunction. Transcriptomic analysis identifies PPARα as the fatty acid sensor mediating the immunosuppressive effects of TDEs on DCs. PPARα blockade effectively restores DC function and enhances the efficacy of immunotherapy.

Yin et al., 2020, Cell Reports *33*, 108278
October 20, 2020 © 2020 The Authors.
https://doi.org/10.1016/j.celrep.2020.108278

CellPress

Cell Reports

CellPress
OPEN ACCESS

Article

PPARα Inhibition Overcomes Tumor-Derived Exosomal Lipid-Induced Dendritic Cell Dysfunction

Xiaozhe Yin,[1,2,6] Wenfeng Zeng,[1,2,6,*] Bowen Wu,[1,2,3] Luoyang Wang,[4] Zihao Wang,[1,2] Hongjian Tian,[1,2] Luyao Wang,[1,2] Yunhan Jiang,[5] Ryan Clay,[5] Xiuli Wei,[1] Yan Qin,[1] Fayun Zhang,[1] Chunling Zhang,[1] Lingtao Jin,[5,*] and Wei Liang[1,2,7,*]

[1]Protein and Peptide Pharmaceutical Laboratory, Institute of Biophysics, Chinese Academy of Sciences, Beijing 100101, China
[2]University of Chinese Academy of Sciences, Beijing 100864, China
[3]Laboratory of Proteomics, Institute of Biophysics, Chinese Academy of Sciences, Beijing 100101, China
[4]Department of Chemical Engineering, Tsinghua University, Beijing 100084, China
[5]Department of Anatomy and Cell Biology, College of Medicine, University of Florida, Gainesville, FL 32610-3033, USA
[6]These authors contributed equally
[7]Lead Contact
*Correspondence: zengwenfeng@ibp.ac.cn (W.Z.), ljin1@ufl.edu (L.J.), weixx@sun5.ibp.ac.cn (W.L.)
https://doi.org/10.1016/j.celrep.2020.108278

SUMMARY

Dendritic cells (DCs) orchestrate the initiation, programming, and regulation of anti-tumor immune responses. Emerging evidence indicates that the tumor microenvironment (TME) induces immune dysfunctional tumor-infiltrating DCs (TIDCs), characterized with both increased intracellular lipid content and mitochondrial respiration. The underlying mechanism, however, remains largely unclear. Here, we report that fatty acid-carrying tumor-derived exosomes (TDEs) induce immune dysfunctional DCs to promote immune evasion. Mechanistically, peroxisome proliferator activated receptor (PPAR) α responds to the fatty acids delivered by TDEs, resulting in excess lipid droplet biogenesis and enhanced fatty acid oxidation (FAO), culminating in a metabolic shift toward mitochondrial oxidative phosphorylation, which drives DC immune dysfunction. Genetic depletion or pharmacologic inhibition of PPARα effectively attenuates TDE-induced DC-based immune dysfunction and enhances the efficacy of immunotherapy. This work uncovers a role for TDE-mediated immune modulation in DCs and reveals that PPARα lies at the center of metabolic-immune regulation of DCs, suggesting a potential immunotherapeutic target.

INTRODUCTION

Dendritic cells (DCs) are professional antigen-presenting cells and play a pivotal role in orchestrating immune responses against pathogen infection or tumor development (Preynat-Seauve et al., 2006). Tumor-infiltrating DCs (TIDCs) present tumor-associated antigens to effector T cells and facilitate the induction of memory T cells to prevent tumor recurrence (Diamond et al., 2011), as well as enhance the efficiency of checkpoint therapy (Garris et al., 2018). However, various immunosuppressive factors in the tumor microenvironment (TME) undermine DC function by inhibiting DC maturation and antigen presentation and enhancing checkpoint protein expression (Apetoh et al., 2011). Importantly, immune dysfunctional DCs result in uncontrolled tumor progression (Scarlett et al., 2012), indicating that maintaining the immune competence of TIDCs is critical for successful anti-tumor immunity.

Recent studies have shown that metabolic rewiring is strongly connected with the functional states of DCs (Dong and Bullock, 2014; Wculek et al., 2019). A shift toward glycolysis promotes an immunogenic or proinflammatory state in DCs. The use of fatty acids (FAs) as the preferred carbon source with augmented FA oxidation (FAO) favors tolerogenic DCs (Everts and Pearce, 2014; Malinarich et al., 2015; Zhao et al., 2018). However, the contribution of lipid metabolism to the tolerogenic feature of DCs is still under debate. Ferreira et al. (2015) showed that glycolysis instead of FAO is essential for the tolerogenic phenotype of DCs, which was also supported by another study (Dáňová et al., 2015). Other studies have also indicated that FAO, an essentially catabolic process, can impair DC effector functions in the TME (Zhao et al., 2018). Thus, the role of lipid metabolism in regulating DC function, particularly in the TME, is still largely undefined. Interestingly, TIDCs exhibit a "lacy" phenotype featuring highly enriched lipid droplets (LDs), and lipid-laden TIDCs display an impaired potential to present tumor-associated antigens (TAAs) (Ramakrishnan et al., 2014). However, the complex network in the TME that induces lipid-mediated DC immune dysfunction remains largely unknown.

Secreted by nearly all types of cells, exosomes contain signaling molecules, such as proteins, nucleic acids, and lipids, and are increasingly considered an important mediator of intercellular communication. Tumor-derived exosomes (TDEs) have

This is an open access article under the CC BY license (http://creativecommons.org/licenses/by/4.0/).

been recognized increasingly as a major immunosuppressive factor in the TME (Milane et al., 2015; Whiteside, 2016). Previous studies have been focused on suppressive mechanisms of mRNAs or microRNAs (miRNAs) encapsulated in TDEs; however, little is known about the relationship between lipid composition in TDEs and the immune cells that engulf them, especially TIDCs. Lipidomes of exosomes derived from hepatocellular carcinoma cells and human bone marrow-derived mesenchymal stem cells have been shown to contain enriched glycolipid, FAs, and phosphatidylserine (Haraszti et al., 2016). Among enriched lipid species, FAs are essential substrates for energy production and serve as building blocks for most newly synthesized lipid components. Nevertheless, excess FAs in the cytoplasm can also negatively affect the physiological functions of the cell (Cabodevilla et al., 2013; Rambold et al., 2015).

In the present study, we hypothesize that lipid-laden TIDCs are induced by TDEs. We uncover that TDE-derived FAs contribute to lipid accumulation (mainly in the form of LDs) and dysfunction of TIDCs. Mechanistically, the engulfment of TDEs by DCs upregulates the expression of peroxisome proliferator-activated receptor α (PPARα), a master regulator involved in the metabolism of lipids, carbohydrates, and amino acids. In response to FAs from TDEs, PPARα activates FAO and induces immune dysfunctional DCs. Importantly, the inhibition of PPARα effectively corrected the immune dysfunction of TIDCs and enhanced the anti-tumor efficacy of immunotherapies. Collectively, our findings indicate that TDEs, as FA carriers, negatively regulate DCs and targeting PPARα could be a promising anti-tumor strategy and of great therapeutic benefit.

RESULTS

TDEs Induce Lipid-Laden DCs

As previously reported (Calder, 2010; Cubillos-Ruiz et al., 2015; Herber et al., 2010), we initially observed that tumor-infiltrating DCs were characterized by lipid accumulation and defective in priming cytotoxic CD8$^+$ T cells (Figures 1A–1C and S1A). We also observed LD formation in bone marrow-derived DCs (BMDCs) co-cultured with tumor cells (Figure 1D) or tumor culture medium (TCM) *in vitro* (Figure 1E). It was suggested that tumor-derived factors may be responsible for the induction of lipid-laden TIDCs. The specific factors that induce lipid-laden TIDCs, however, remain elusive. To further identify the critical factors mediating this effect, we divided TCM from TC-1 cervical cancer cells with a 100-kDa and a 30-kDa molecular weight cutoff filter, respectively. Then, we treated BMDCs with the three obtained fractions—<30 kDa (fraction S), between 30 and 100 kDa (fraction M), and >100 kDa (fraction L)—followed by determining the amount of neutral lipids in BMDCs using Bodipy 493/503 staining. Interestingly, fraction L increased the neutral lipid content in BMDCs as effectively as TCM (Figure 1F). These data suggest that super-molecules or subcellular organelles in TCM play an important role in inducing lipid accumulation in BMDCs. However, we observed that fraction S and fraction M also increased the lipid level, although to a lesser degree (Figure 1F), indicating that small molecules or soluble proteins may contribute to this effect as well.

TDEs are important subcellular membrane particles with a diameter ranging from 30 to 120 nm, and can trigger immunosuppression by communicating with various immune cells (Fonseca et al., 2016). Thus, we sought to determine whether the TDEs in fraction L are responsible for lipid accumulation in BMDCs. To test our hypothesis, we isolated TDEs from fraction L of the TCM via centrifugation. The size and morphology of TC-1-derived exosomes were characterized using transmission electron microscopy (TEM; Figure 1G), and western blot analysis of the exosome marker proteins (ALIX, HSP70, TSG101, CD9, and CD81) (Figures 1H and S1B). We observed that exosomes alone increased the intracellular lipid in BMDCs, and the exosome-free part did not (Figure 1I). Moreover, exosomes secreted from cancer cells from different tumor types (4T1 and B16/F10) also triggered excess lipid accumulation in BMDCs (Figure S1C).

To better understand the causal relationship between TDEs and the lipid accumulation in TIDCs, we generated the GFP-CD9-tumor cells (TC-1 and MC38), which endowed exosomes labeled with GFP and enabled us to trace the TDEs *in vivo* (Figures 1J and S1D). In mice injected with GFP-CD9-TC-1 tumor cells, we detected the percentage of GFP$^+$ cells and measured the intracellular lipid content in TIDCs (Figure 1K). The results showed that only TIDCs that captured GFP-labeled TDEs were lipid abundant and immune dysfunctional, whereas GFP$^-$ TIDCs were not (Figures 1L and 1M). In addition, we found that tumor-associated macrophages (TAMs), another antigen-presenting cell, also captured fluorescence-labeled TDEs at the tumor site and showed a higher lipid content (Figures 1K and S1E). Moreover, we treated BMDCs with GFP-labeled TDEs *in vitro*, and found that BMDCs that took up GFP-TDEs showed an increase in LDs (Figures S1F and S1G). To further substantiate this finding, we injected PKH67-labeled TDEs directly into the tumor (MC38) and measured the percentage of PKH67$^+$ cells and the intracellular lipid content in TIDCs, TAMs, monocytes, and neutrophils (Figure S1H). Results showed that all of the cell populations could uptake PKH67-labeled exosomes, and that much larger amounts of exosomes were engulfed by TIDCs and TAMs than by neutrophils and monocytes (Figure S1I). Also, similar to GFP-labeled exosomes, both TIDCs and TAMs that took up PKH67-labeled exosomes (PKH67$^+$ cells) had more intracellular lipid content (Figure S1J). However, the involvement of macrophages in TDEs that induced immunological alteration would be investigated in later studies (see Figure S6).

To further confirm that TDEs are able to interfere with the immune function of DCs directly, we injected purified PKH67-labeled TDEs into the footpads of wild-type (WT) C57BL/6 mice and measured the phenotypic changes in DCs in popliteal lymph nodes (near) and inguinal lymph nodes (far). The results showed that DCs in lymph nodes near the footpads engulfed more TDEs (Figure S1K), had more accumulated lipids (Figure S1L), and were less capable of priming CD8$^+$ T cells than those in lymph nodes far from the footpads (Figure S1M and S1N), which may be due to their different accessibility to TDEs. Collectively, these data suggest that TDEs are crucial for promoting lipid accumulation in TIDCs.

TDEs Drive DC Immune Dysfunction

Previous studies have suggested that TIDCs associated with lipid accumulation are defective in priming CD8$^+$ T cells (Cubillos-Ruiz et al., 2015; Herber et al., 2010). Thus, it is plausible

CellPress
OPEN ACCESS

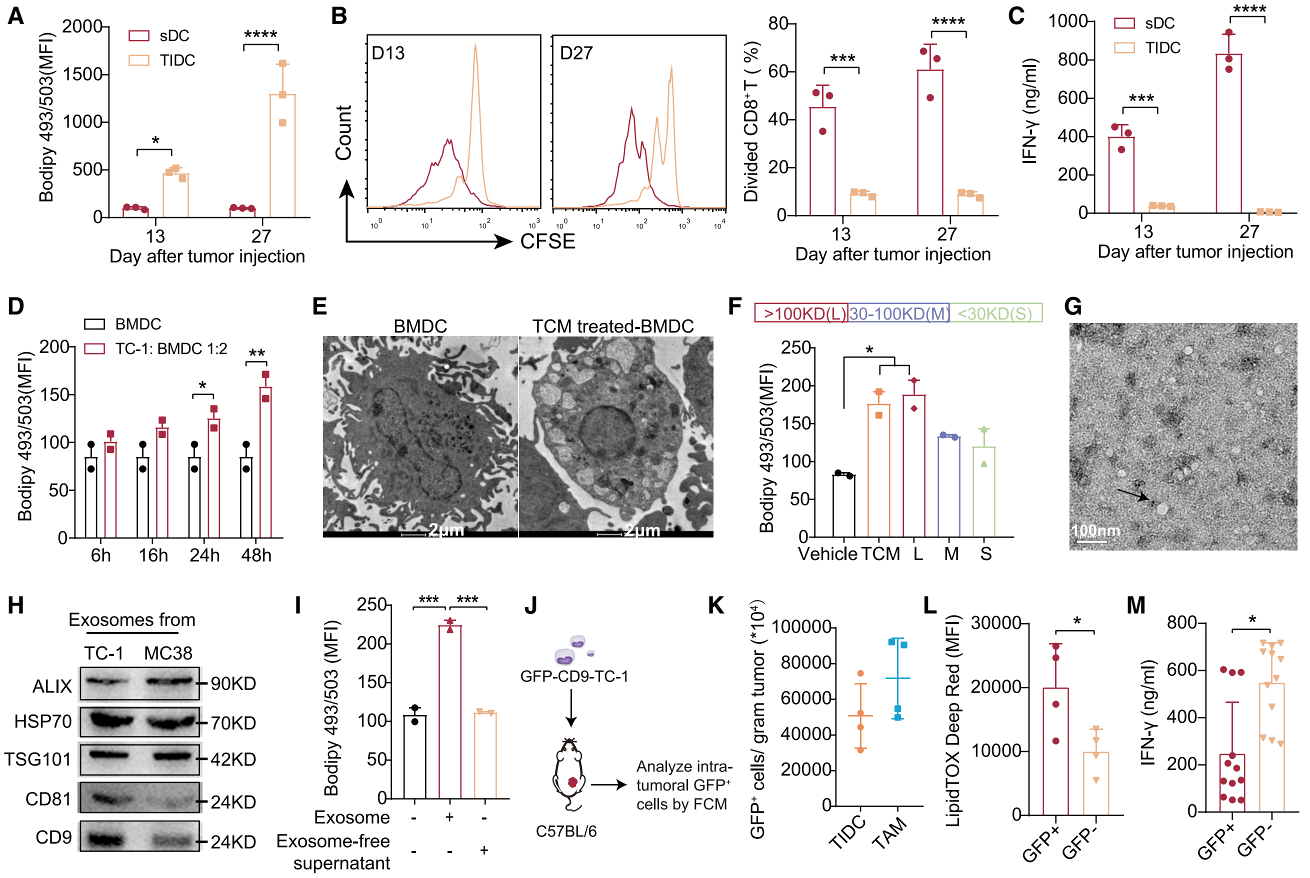

Figure 1. Tumor-Derived Exosomes (TDEs) Promote Lipid Accumulation in Dendritic Cells (DCs)

In the MC38-OT I tumor model, DCs (CD45$^+$MHC II$^+$CD11c$^+$ F4/80$^-$) in spleen (sDCs) and in tumor (TIDCs) were analyzed on days 13 and 27 (n = 3 per group).

(A) Intracellular lipid in sDCs and TIDCs.

(B and C) The proliferation and function of OT I CD8$^+$ T cells primed by sDCs or TIDCs were evaluated by CFSE dilution (B) and IFN-γ production (C).

(D) Intracellular lipid levels in BMDCs co-cultured with or without TC-1 tumor cells.

(E) Lipid droplets in BMDCs were photographed by transmission electron microscopy (TEM). Scale bar, 2 μm.

(F) Tumor cell medium (TCM) was separated with an MWCO filter (30 and 100 kDa cutoff). Intracellular lipid levels in BMDCs after incubation with different fractions for 24 h.

(G) TEM analysis of exosomes secreted by TC-1 cancer cells. Scale bar, 100 nm.

(H) Western blot analysis of exosome markers.

(I) Intracellular lipid levels in BMDCs treated with tumor-derived exosomes (400 μg/mL) or exosome-free supernatant for 24 h.

(J) Scheme of analysis.

(K) GFP$^+$ cells/gram tumor tissue of TIDCs and TAMs were measured (n = 4 per group).

(L) Intracellular lipid levels in TIDCs.

(M) Isolated GFP$^+$ and GFP$^-$ TIDCs were co-cultured with OT I CD8$^+$ T cells for 3 days to detect IFN-γ production by ELISA. Each dot represents technical repeats.

*$p < 0.05$; **$p < 0.01$; ***$p < 0.001$; ****$p < 0.0001$. (A)–(D) were analyzed with 2-way ANOVA. (F) and (I) were analyzed with 1-way ANOVA. (K)–(M) were analyzed with 2-tailed t test. Error bars represent SEMs. The error bars represent SDs for (C) and (M), and SEM for all of the others. Each dot represents an individual mouse in (A)–(D), (F), (I), and (K)–(M). Representative of 3 independent experiments in (F) and 2 independent experiments in (I).

See also Figures S1 and S6.

that TDEs not only induce lipid accumulation in DCs but also negatively affect the priming function of DCs. Data analyses on TDE-treated BMDCs revealed that TDEs significantly upregulated the expression of inhibitory checkpoint proteins such as programmed cell death ligand 1 (PD-L1) and signal regulatory protein α (SIRPα) (Figure 2A), and increased the secretion of immunosuppressive factor transforming growth factor-β (TGF-β) (Figure 2B). However, TDEs also slightly elevated molecules with immunostimulatory capabilities in response to T cells (Figure S2A). Next, we examined the ability of BMDCs to present antigens by using a 25-d1.16 antibody, which recognizes the ovalbumin (OVA)-derived epitope bound to H2K^b. We also incubated CD8$^+$ T cells from OTI transgenic mice with BMDCs pretreated with OVA in the presence or absence of TDEs (at a safe concentration; Figure S2B) to detect the ability of BMDCs to prime CD8$^+$ T cells. The expression of peptide major

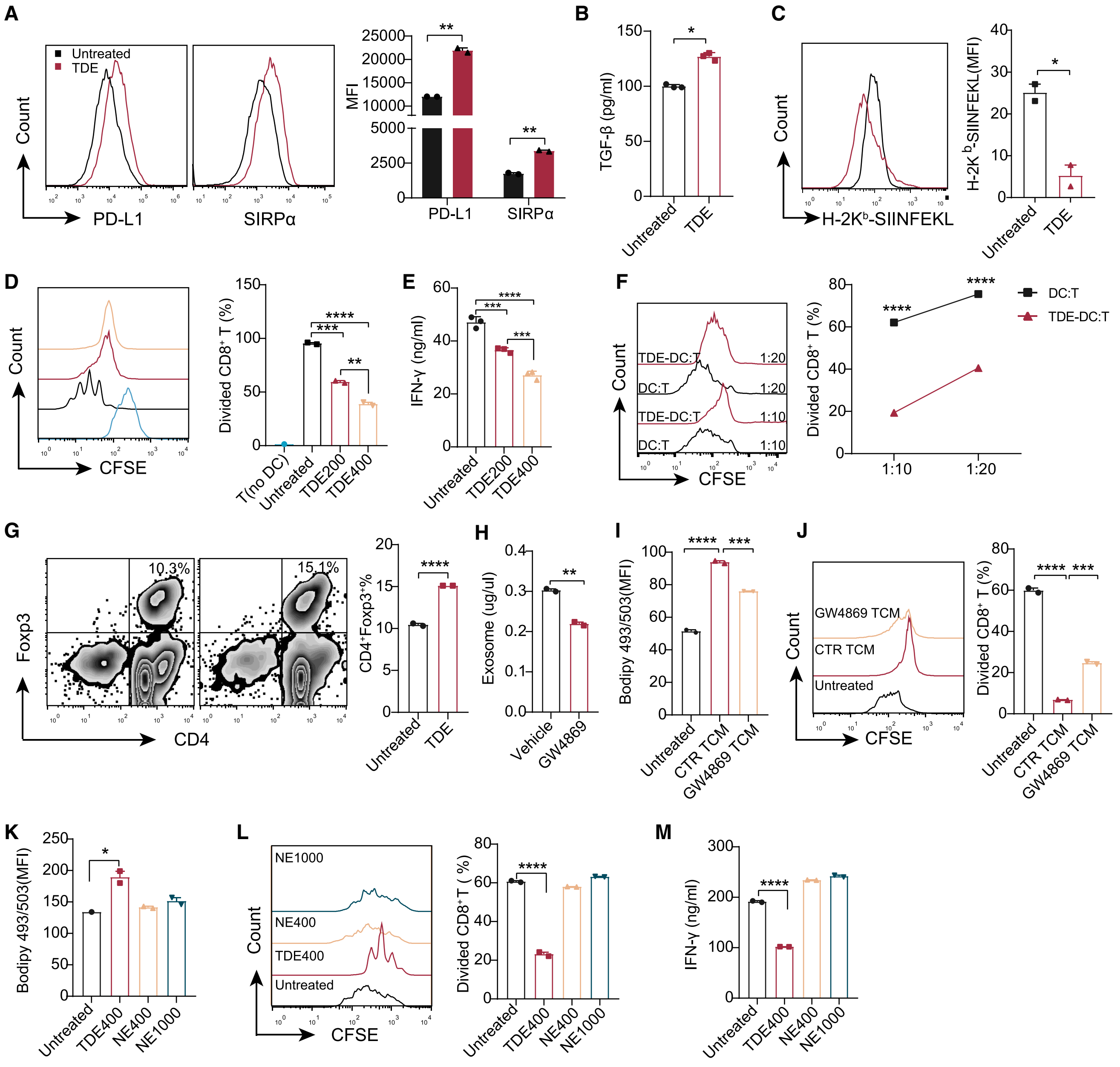

Figure 2. TDEs Mediate DC Dysfunction

BMDCs were treated with or without TDEs (400 μg/mL) for 24 h.

(A) Fluorescence-activated cell sorting (FACS) analysis of PD-L1 and SIRPα.

(B) Secreted TGF-β was analyzed by ELISA.

(C) BMDCs were treated with 2 mg/mL OVA in the presence or absence of TDEs (200 μg/mL) for 24 h. H-2K^b-SIINFEKL on the surface of BMDCs was assessed by the 25.D1 antibody.

(D and E) BMDCs were treated with 2 mg/mL OVA in the presence or absence of TDEs for 48 h, followed by antigen removal, and co-cultured with OT I CD8$^+$ T cells for 3 days. Proliferation (D) and IFN-γ production (E) of OT I CD8$^+$ T cells were analyzed. T, undivided OT I cells as a negative control.

(F) Inhibition of CD8$^+$ T cell proliferation by TDE-treated BMDCs.

(G) Generation of Tregs (CD4$^+$Foxp3$^+$) by TDE-treated BMDCs.

(H) Quantitative analysis of exosomes in the supernatant of MC38 cells treated with DMSO or 10 μM GW4869 (48 h).

(I and J) BMDCs were treated with the TCM collected in (H) for 24 h. The intracellular lipid level (I) and proliferation of OT I CD8$^+$ T cells (J) were analyzed.

(K–M) BMDCs were treated with TDEs (400 μg/mL) or non-cancerous cell-derived exosomes (NEs, 400 or 1,000 μg/mL) for 24 h. The intracellular lipid levels in BMDCs (K), proliferation (L), and IFN-γ production (M) of CD8$^+$ T were analyzed.

(legend continued on next page)

histocompatibility complex I (pMHC I) and the carboxyfluorescein succinimidyl ester (CFSE) dilution assay showed that TDE-treated BMDCs were less capable of presenting antigen, no matter the length of antigen (OVA [Figure 2C] or $OVA_{257-264}$ and $OVA_{250-264}$ [Figure S2C]), inducing proliferation (Figures 2D and S2D), and promoting interferon-γ (IFN-γ) production in OTI $CD8^+$ T cells (Figure 2E). Moreover, we found that TDE-treated BMDCs without antigens could directly suppress the proliferation of $CD8^+$ T cells (Figure 2F) and induce the generation of regulatory T cells (Tregs) (Figure 2G). These profiles strongly suggest an immune dysfunctional state in TDE-treated DCs. To further confirm that TDEs contribute to DC dysfunction, we incubated BMDCs with TCM treated with or without GW4869 (Yang et al., 2018), an nSMase inhibitor that reduces exosome secretion (Figure 2H). Lipid level and a CFSE dilution assay showed that GW4869-treated TCM could partially restore both lipid accumulation and the defective T cell priming capability of DCs (Figures 2I and 2J).

In addition to cancer cells, other cells in the TME may produce exosomes and affect DC function. To rule out this possibility, *in vitro* we collected the exosomes from NIH 3T3 cells, a non-cancerous cell line with a fibroblast phenotype (Suvarna et al., 2019). We treated BMDCs with equal amounts of exosomes derived from tumor cells or NIH 3T3 cells and measured their change in intracellular lipids and T cell priming capability. As the data show, exosomes from non-cancerous cell-derived exosomes (NEs) had little effect on the induction of lipid accumulation in BMDCs (Figure 2K) and did not impede the capability of BMDCs to prime $CD8^+$ T cells (Figures 2L and 2M), even when the concentration of NEs exceeded TDEs. Collectively, these data demonstrate that TDEs drive DC immune dysfunction.

FAs in TDEs Induce DC Immune Dysfunction

Given the different effects on DCs between tumor-derived and NEs, we further investigated the underlying mechanism for the increased lipid level and immune dysfunction in TDE-treated DCs. Flow cytometry analysis showed that time- and concentration-dependent lipid accumulation occurred as soon as BMDCs internalized TDEs (Figures S3A and S3B), which was also confirmed by Opera Phenix High Content Screening analysis (Figures 3A and 3B; Videos S1 and S2). The correlation between the concentration of TDEs and the lipid level in DCs prompted us to ask whether increased neutral lipids in DCs were derived from TDEs, and whether NEs contained smaller amounts of lipids so that they could not induce lipid-laden DC. To address this question, we performed a lipidomic analysis on NEs, TDEs, and TDE-treated BMDCs. Consistent with previous reports (Haraszti et al., 2016), TDEs did contain abundant FA species compared to NEs (Figure 3C). In addition, nearly all of the FA species enriched in TDEs were correspondingly increased in TDE-treated BMDCs compared to the control group, and the most abundant ones were long-chain FAs (LCFAs, C16–C22) either saturated or unsaturated (Figure 3D). Importantly, FAs contained in TDEs and the TDE-treated BMDCs are strongly positively correlated (Figure 3E). The highly correlated LCFAs among them are C16:0 (palmitic acid), C18:0 (stearic acid), C18:1n9 (oleic acid), and C20:4n6 (arachidonic acid) (Figure 3E). To further illustrate the crucial role of TDE-derived FAs for lipid accumulation in DCs, we prepared a FAs mixture (FA mix) with the same molar concentration of these four LCFAs as found in TDEs (namely, C16:0, C18:0, C18:1n9, and C20:4n6). The results showed that both FA mix and TDEs could induce lipid accumulation in BMDCs and suppress proliferation as well as IFN-γ production in $CD8^+$ T cells (Figures 3F–3H). These findings explain why exosomes from non-cancerous cells had no effect on the lipid level in DCs and suggest that the FAs from TDEs, rather than from NEs, cause intracellular lipid accumulation in DCs.

The FAs required for triglyceride synthesis come from two sources: exogenous FAs (in this case, FAs from TDEs) and endogenous synthesized FAs (Figure 3I). Therefore, we sought to determine which FA source is responsible for TDE-induced triglyceride accumulation. First, to block exogenous TDEs uptake, various endocytosis inhibitors were used while DCs internalized TDEs (Table S1) (Mulcahy et al., 2014; Svensson et al., 2013). The results revealed that only chloropromazine (CPZ) blocked the internalization of TDEs as effectively as low temperature (4°C) did (Figure S3C). Second, we used CPZ to block TDE uptake and used specific inhibitors to block key steps in *de novo* synthesis pathways, such as ACC (acetyl-coenzyme A [CoA] carboxylase) inhibitor 5-tetradecyloxy-2-furoic acid (TOFA) and fatty acid synthase (FASN) inhibitor C75. Neither TOFA nor C75 reduced the elevation of lipid content induced by TDEs (Figure 3J), indicating that endogenous FAs are dispensable for TDE-induced lipid accumulation. However, triacsin C (an inhibitor of long-chain acyl-CoA synthetase [ACSL]) completely blocked lipid accumulation in BMDCs, similar to CPZ (Figure 3J). Coincidentally, TDEs contained LCFAs that were substrates of ACSL, which catalyzes FAs to form acyl-CoAs during triglyceride synthesis (Yan et al., 2015). Thus, these data demonstrate that lipid accumulation in TDE-treated DCs is the result of the uptake of TDE-derived FAs rather than TDE-induced *de novo* FA synthesis. Collectively, our data suggest that FAs contained in TDEs directly induce lipid accumulation and immune dysfunction in DCs.

TDE-Derived FAs Activate PPARα Signaling to Suppress DC Priming Ability

To explore the molecular mechanisms by which TDEs trigger lipid accumulation and immune dysfunction in DCs, we performed global transcriptomic profiling by RNA sequencing (RNA-seq) in TDE-treated DCs, NE-treated DCs, and control (CTR) DCs. Gene pathway analysis, with a particular focus on metabolic pathways, revealed that DCs with TDE treatment highly expressed the genes responsible for lipid metabolism compared with CTR DCs (Figure 4A), which was also identified by proteomics (Figure S4A). Furthermore, we specifically

*$p < 0.05$; **$p < 0.01$; ***$p < 0.001$; ****$p < 0.0001$. (B), (C), (G), and (H) were analyzed with 2-tailed t test. (A) was analyzed with 2-way ANOVA. The other data were analyzed with 1-way ANOVA. The error bars represent SEMs. The error bars of (B), (E), and (M) represent SDs. Representative of 4 independent experiments in (D) and (E), 3 independent experiments in (A) and (K)–(M), and 2 independent experiments in (B), (C), (F), and (G).
See also Figure S2.

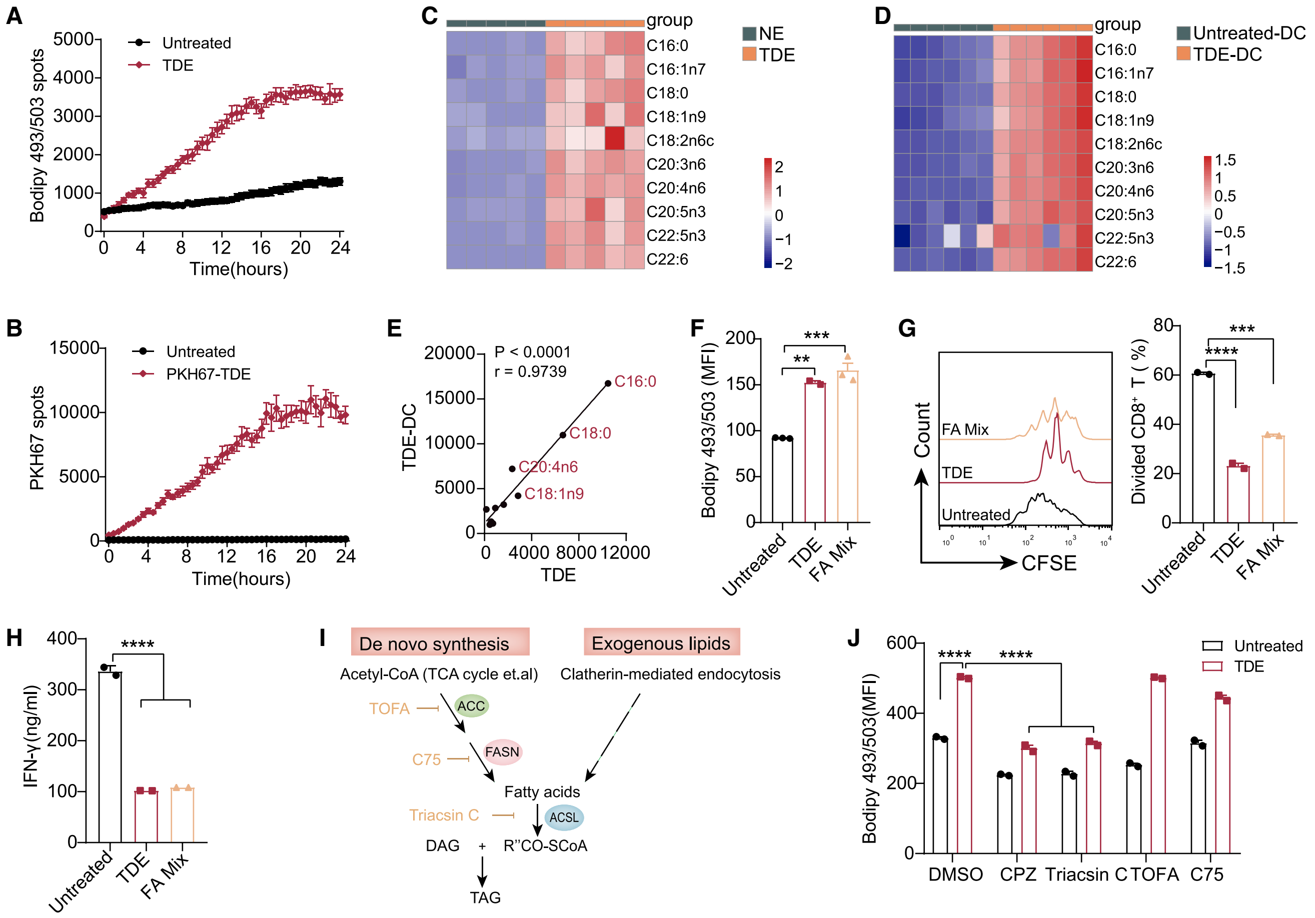

Figure 3. Fatty Acids (FAs) Derived from TDEs Induce Intracellular Lipid Accumulation in DCs

(A) The real-time intracellular lipid content change was detected by Bodipy 493/503 and recorded every 30 min by Opera Phenix High Content Screening System (also shown in Video S1).

(B) The uptake of TDEs was indicated by PKH67 and recorded every 30 min by Opera Phenix High Content Screening System (also shown in Video S2).

(C) Lipidomics analysis of TDEs and NEs.

(D) Lipidomics analysis of BMDCs cultured with or without TDEs (400 μg/mL) for 48 h.

(E) Correlation analysis of FAs between TDEs and TDE-treated DCs.

(F) Intracellular lipid levels in BMDCs treated with TDEs (400 μg/mL) or with FA mix (C16:0 30 μM, C18:0 15 μM, C18:1n9 15 μM, and C20:4n6 20 μM) for 24 h.

(G and H) Proliferation (G) and IFN-γ production (H) of $CD8^+$ T were analyzed.

(I) Schematic diagram showing the triglyceride synthesis. ACC, acetyl-CoA carboxylase; ACSL, long-chain acyl-CoA synthetase; DAG, diacylglycerol; FASN, FA synthase; TAG, triglyceride.

(J) Intracellular lipid levels in BMDCs cultured with or without TDEs (400 μg/mL) for 24 h in the presence of DMSO, chloropromazine (CPZ) (40 μM), triacsin C (15 μM), TOFA (5 μg/mL), and C75 (30 μM).

**p < 0.01; ***p < 0.001; ****p < 0.0001. (J) was analyzed with 2-way ANOVA. Other data were analyzed with 1-way ANOVA. The error bars represent SEMs. The error bars of (H) represents SDs. Representative of 3 independent experiments in (F)–(H), and (J).

See also Figure S3.

assessed changes in the expression of genes related to lipid metabolism and found that TDE-treated DCs displayed increased expression in these genes compared to the other two groups (Figure 4B). According to the lipidomic analysis, the same gene expression changes were observed in both NE-treated and CTR DCs (Figure 4B), further suggesting the crucial role of TDE-derived FAs. Furthermore, we found that gene enrichment pathways, such as lipid storage and FA metabolism, were substantially related to PPARs (Figure 4C). PPARs are ligand-activated transcription factors that regulate the expression of numerous genes involved in lipid metabolism, including genes encoding LD-associated proteins and lipogenic and lipolytic enzymes (Rubinow et al., 2013). The fluctuation of intracellular concentration of these FAs can influence PPAR-dependent gene regulation (Wahli and Michalik, 2012; Zúñiga et al., 2011). Transcriptional analysis revealed that PPARα and its several downstream genes, such as Ppargc1a, Acsls, Cpts, and Dgat2, which are involved in LD generation and FAO, were upregulated in DCs with TDE treatment, while the expression of PPARγ was downregulated (Figure 4D). NE-treated DCs,

CellPress
OPEN ACCESS

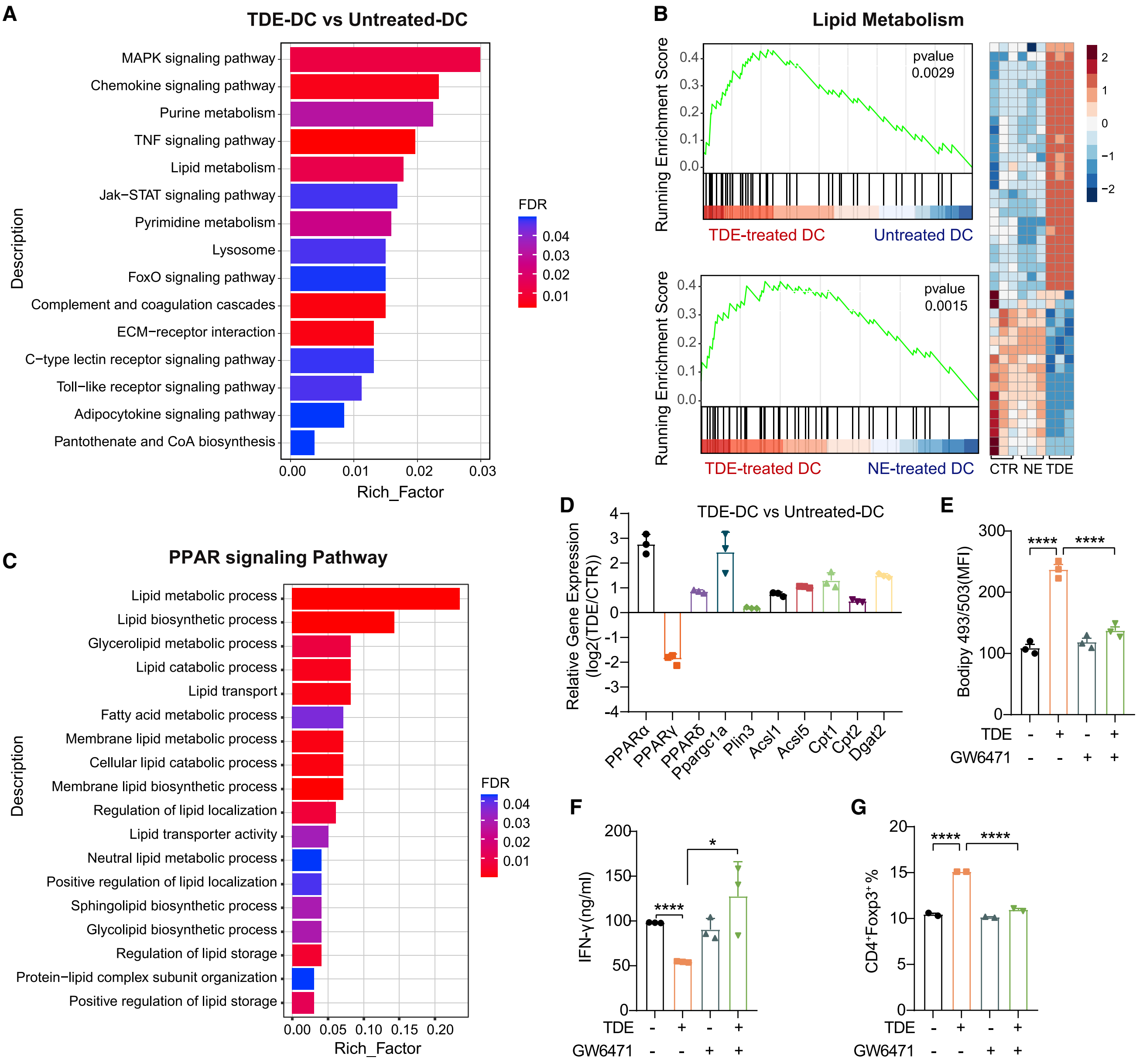

Figure 4. TDE-Mediated PPARα Activation Triggers DC Lipid Accumulation and Dysfunction

(A) Pathways enrichment analysis (Kyoto Encyclopedia of Genes and Genomes [KEGG]) in TDE-treated DCs compared with control DCs.

(B) GSEA enrichment plots of lipid metabolism pathways in TDE-treated DCs compared with control DCs (top) or NE-treated DCs (bottom). Red represents a high expression level and blue indicates a low expression level. The heatmap represents each involved gene (right).

(C) Pathway enrichment analysis by Gene Ontology (GO) focusing on PPAR signaling pathway between TDE-treated DCs and untreated DCs.

(D) Expressions of the indicated transcripts were assessed by RNA-seq.

(E) BMDCs were incubated with or without TDEs (400 μg/mL) in the presence or absence of GW6471 (15 μM) for 48 h. Lipid content was assessed.

(F) The production of IFN-γ by $CD8^+$ T cells was assessed.

(G) Generation of Tregs ($CD4^+Foxp3^+$) was analyzed by flow cytometry.

*$p < 0.05$; ****$p < 0.0001$. Data were analyzed with 1-way ANOVA. The error bars represent SEMs. The error bar of (F) represents SDs. Representative of 3 independent experiments in E) and (F), and 2 independent experiments in (G).

See also Figure S4.

however, showed different expression of PPARα and PPARγ (Figure S4B), indicating a different regulatory mechanism between TDEs and NEs. Importantly, DCs in popliteal lymph nodes that took up TDEs also showed an elevated expression of PPARα, but not PPARγ or PPARδ (Figure S4C). Then, we investigated whether PPARα was crucial for TDE-induced adverse

effects on BMDCs. Bodipy 493/503 staining revealed that PPARα inhibitor GW6471 significantly reduced TDE-induced lipid accumulation (Figure 4E). We further treated OVA-primed BMDCs with TDEs in the presence or absence of GW6471 before they were co-cultured with OT I CD8$^+$ T cells. Strikingly, GW6471 abrogated the TDE-mediated suppressive effect on BMDCs, leading to enhanced IFN-γ production from T cells (Figure 4F), whereas PPARδ inhibitor GSK3787 had no effect (Figures S4D–S4F). Furthermore, PPARα inhibition decreased the TDE-induced Treg induction in BMDCs (Figure 4G). These data reveal that PPARα plays a potent regulatory role in TDE-induced immune dysfunction of DCs.

PPARα-Mediated FAO Is the Key Pathway Mediating DC Dysfunction

To uncover the mechanism by which PPARα signaling regulates DC function, we consider whether PPARα controls TDE uptake by DCs. However, the inhibition of PPARα by GW6471 significantly reduced TDE-induced lipid accumulation but had no impact on TDE uptake (Figure S5). Thus, we hypothesized that TDEs reprogram DC metabolism by activating PPARα. To process FAs by β-oxidation, cells need more mitochondria (Kelly and Scarpulla, 2004; Yao et al., 2019). Confocal imaging and flow cytometry analysis showed that TDEs increased mitochondrial mass in BMDCs (Figures 5A and 5B). Moreover, we found that TDE-treated BMDCs and TIDCs both had an increased mitochondrial membrane potential (TMRM) (Figures 5B and 5C), suggesting that TDE-engulfed DCs may require more mitochondria and higher mitochondrial capacity to process excess FAs. Next, we used seahorse extracellular flux analysis to measure the oxygen consumption rate (OCR) and extracellular acidification rate (ECAR), which reflect the activity of oxidative phosphorylation (OXPHOS) and glycolysis, respectively. Basal and maximum OCR were markedly increased in TDE-treated BMDCs (Figures 5D and 5E), indicating the robust enhancement of OXPHOS. The data of the ECAR demonstrated that TDEs decreased the rate of glycolysis (Figure 5F), which is further supported by the decrease in lactate production in TDE-treated BMDCs (Figure 5G). Strikingly, the inhibition of PPARα by GW6471 treatment restored these changes. Direct measurement of the FAO capacity in BMDCs further demonstrated that TDE treatment promoted FAO activity, which was also abrogated by GW6471 (Figures 5H and 5I). These data suggest that TDEs shift the metabolism of DC from a glycolytic state toward OXPHOS in a PPARα-dependent manner.

In fact, PPARα controls FA metabolism via two distinct pathways: catalyzing FAs to form acyl-CoA for LD generation by Acsl and transporting FAs into mitochondria for β-oxidation by Cpt1 (Grevengoed et al., 2015). Interestingly, the mobilization of FAs released from LDs to mitochondria is crucial for cellular survival during nutrient stress (Rambold et al., 2015). Accordingly, we investigated whether the inhibition of Ascl or Cpt1 could reverse DC function. As expected, the sole inhibition of Acsl or Cpt1 with their specific antagonist partially restored DC function, while blocking both of these pathways relieved the immune function of TDE-treated DCs, which is consistent with GW6471 (Figures 5J and 5K). These results provide a molecular basis for the TDE-mediated lipid metabolic reprogramming of DCs and demonstrate that PPARα plays an essential role in the TDE-induced immune dysfunction of DCs.

PPARα Inhibition Enhances the Anti-tumor Efficacy of Immunotherapies

Our observation that blocking PPARα restored DCs immune function *in vitro* prompted us to examine whether PPARα blockade decreased the lipid content of TIDCs or altered the ability of TIDCs to prime CD8$^+$ T cells and promote anti-tumor immunity. Therefore, we isolated TIDCs from the MC38-OT I cancer model and performed *ex vivo* functional assays. Consistent with prior results in BMDCs, the administration of PPARα inhibitor GW6471 reduced intracellular lipid accumulation in TIDCs (Figure 6A) and restored the function of the TIDCs to that of primed T cells (Figures 6B and 6C). Meanwhile, we also used PPARα knockout (KO) mice to verify whether PPARα signaling plays an important role in maintaining the function of TIDCs *in vivo*. By comparing MC38-OT I-bearing PPARα$^{-/-}$ mice to their WT littermates, we found that PPARα deficiency resulted in decreased lipid content (Figure 6D), a higher ratio of antigen-specific tet$^+$ CD8$^+$ T cells (Figure 6E), and improved function of CD8$^+$ T cells (Figure 6F). In addition, we found that tumor growth in PPARα$^{-/-}$ mice was slower than in WT mice (Figure 6G).

In accordance with previous findings that TDEs substantially increased the expression of PD-L1 on DCs (Figure 2A), we hypothesized that PPARα inhibition combined with PD-L1 blockade may initiate more powerful anti-tumor efficacy. Thus, we evaluated a combination therapy using GW6471 and an anti-PD-L1 antibody (Figure 6H). This combination led to a significant regression of MC38-OT I established tumors compared with monotherapies (Figure 6I). Strikingly, the combination therapy further resulted in an increased tumor-free mice ratio (Figure 6J) and a significantly longer lifespan compared with mice that received monotherapies (Figure 6K). GW6471 combined with PD-L1 blockade strongly increased the number of tumor antigen-specific CD8$^+$ T effector cells (OT I tetramer$^+$) (Figure 6L) and highly boosted the production of granzyme B (GrmB), perforin, and IFN-γ from CD8$^+$ T cells (Figure 6M).

Finally, to further evaluate the therapeutic efficacy of PPARα inhibition combined with immunotherapy, we examined whether GW6471 could enhance the anti-tumor efficacy of a therapeutic vaccine. Therapeutic vaccines, which are a potential immunotherapeutic approach for the treatment of cancer, rely strongly on the competency of DCs to function (Liu et al., 2017; Palucka and Banchereau, 2013). We have recently developed a nanosize E7-associated peptide-encapsulated vaccine, which was designed to treat cervical cancer (TC-1), and harbors E7 antigen derived from human papillomavirus (HPV) (Liu et al., 2017). We found that treating TC– established tumors with GW6471 strongly increased the anti-tumor efficacy of the E7 vaccine (Figures 6N and 6O). Importantly, PPARα inhibition also enhanced the anti-tumor efficacy of PD-1 antibody in the less immunogenic B16/F10 melanoma model (Figure S6A).

Ablation of DC Abrogates the Therapeutic Efficacy of Immunotherapy Combined with PPARα Blockade

The improved outcome of MC38-OT I tumors treated with GW6471-PD-L1 antibody combination therapy prompted us to

CellPress
OPEN ACCESS

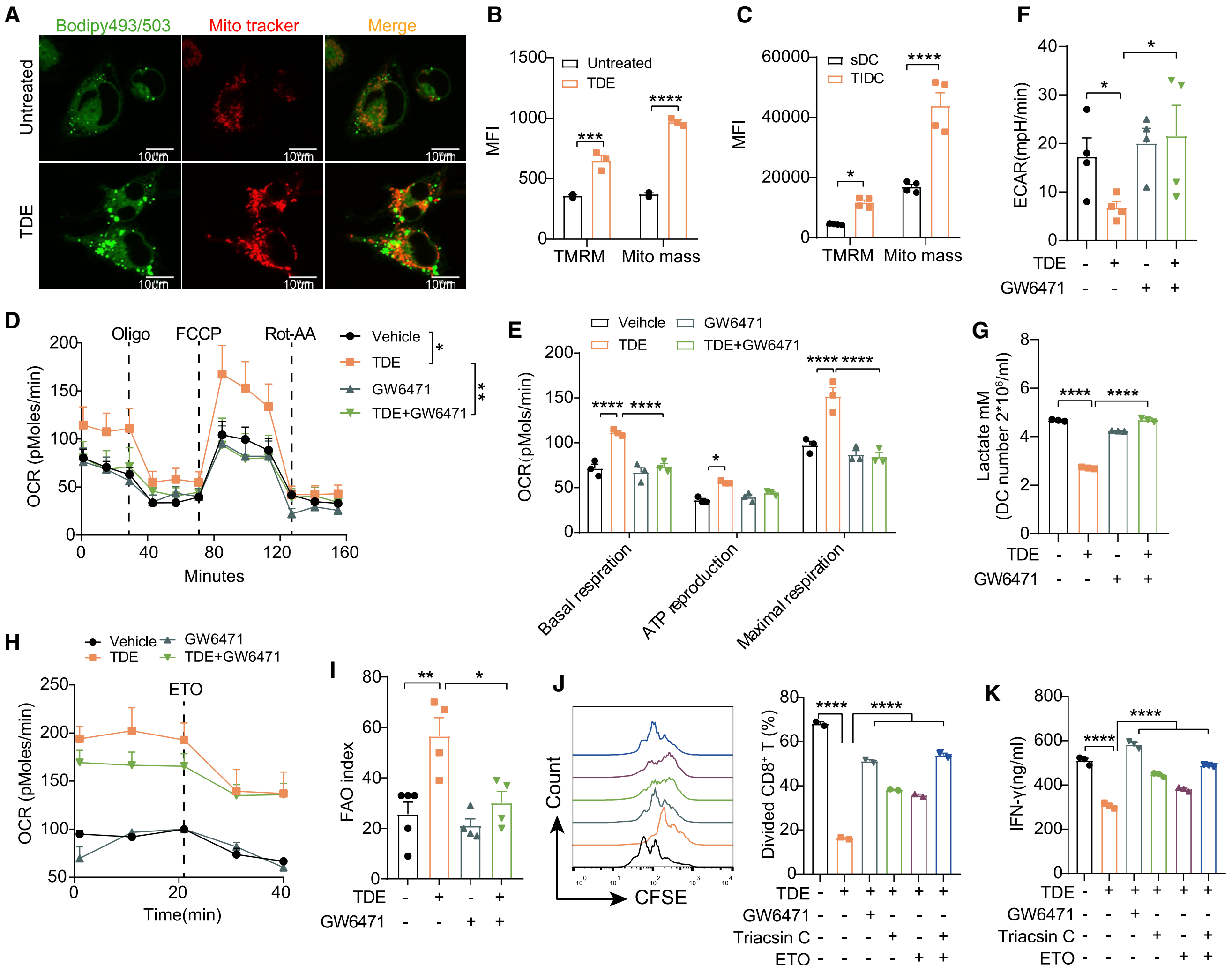

Figure 5. PPARα Activation Reprograms the Lipid Metabolism of DCs

(A) Representative confocal images show the intracellular lipid level and mitochondrial mass of BMDCs treated with or without 400 μg/mL TDEs for 24 h. Scale bar, 10 μm.

(B and C) Mitochondrial membrane potential (TMRM) and mitochondria mass were analyzed in BMDCs treated with or without 400 μg/mL TDEs (B), and sDCs versus TIDCs from MC38-OTI tumor-bearing mice (C).

(D–F) BMDCs pre-treated with or without TDEs (400 μg/mL) in the presence or absence of GW6471 (15 μM) for 48 h; the OCR (D) and ECAR (F) were recorded. The basal respiration, ATP reproduction, and maximal respiration (E) were calculated based on the data in (D). TDE + GW6471 indicates BMDCs treated with TDEs and GW6471.

(G) BMDCs were treated as in (D), and the lactate production was measured.

(H) BMDCs were treated as in (D), and the FAO activity in BMDCs was recorded.

(I) The amount of OCR derived from FAO was quantified as the response of BMDCs to ETO (Etomoxir) treatment.

(J and K) BMDCs were treated with 2 mg/mL OVA in the presence or absence of TDEs (400 μg/mL) plus GW6471 (15 μM), Tracsin C (15 μM) or ETO (40 μM) for 48 h, followed by antigen removal and co-culture with OT I CD8$^+$ T cells for another 3 days. Proliferation (J) and IFN-γ production (K) of OT I CD8$^+$ T cells were analyzed.

*$p < 0.05$; **$p < 0.01$; ***$p < 0.001$; ****$p < 0.0001$. (B)–(E) were analyzed with 2-way ANOVA. Other data were analyzed with 1-way ANOVA. The error bars represent SEMs. The error bars of (G) and (K) represent SDs. Representative of 3 independent experiments in (D)–(F) and 2 independent experiments in (B), (C), (G), (H), (J), and (K).

See also Figure S5.

test whether DCs play a major role in this anti-tumor effect. MC38-OT I tumors were injected in CD11c-diphtheria toxin receptor (CD11c-DTR) chimeric mice, which lack CD11c$^+$ DCs under DT administration (Figure 7A). Strikingly, with DC depleted, the tumor did not respond to combination therapy (Figure 7B), suggesting that the induction of the anti-tumoral immune effect in combination therapy is dependent on DCs. To further verify the role of PPARα signaling in regulating DC function, we sought

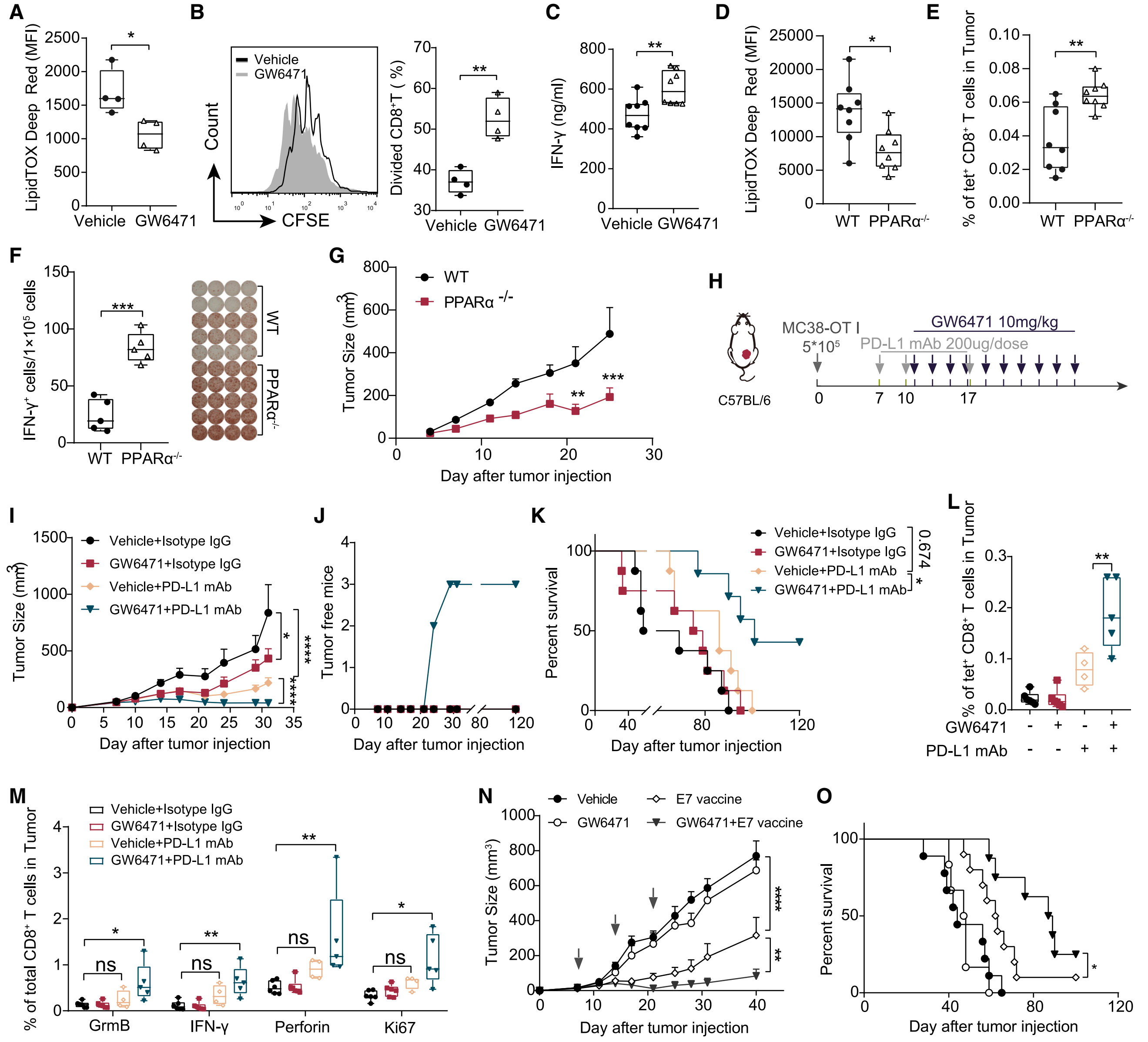

Figure 6. PPARα Inhibition Enhances the Anti-tumor Efficacy of Immunotherapies

(A–C) MC38-OT I-bearing mice were treated with or without 10 mg/kg GW6471 from day 7 after tumor inoculation (every other day, for 6 doses), and tumors were harvested on day 22. The lipid levels of TIDCs (A) were analyzed. The isolated TIDCs from both groups were treated with 2 mg/mL OVA for 48 h, followed by antigen removal and co-culture with OT I $CD8^+$ T cells for another 3 days. Proliferation (B) (n = 4 per group) and IFN-γ production (C) (n = 4 per group) of $CD8^+$ T were analyzed. Each dot represents technical repeats.

(D and E) Lipid levels of TIDCs (D) and the percentage of antigen-specific tumor-infiltrating $CD8^+$ T cells (E) were analyzed in MC38-OT I-bearing WT or $PPAR\alpha^{-/-}$ mice (n = 5 per group).

(F) IFN-γ ELISPOT analysis of tumor-infiltrating $CD8^+$ T cells isolated from MC38-OT I-bearing WT or $PPAR\alpha^{-/-}$ mice (n = 5 per group).

(G) Tumor growth curve between WT or $PPAR\alpha^{-/-}$ mice (n = 5 per group)

(H) Schematic diagram of the combination therapy schedule.

(I–K) Tumor growth curve (I), tumor-free mice numbers (J), and survival curve (K) were shown (n = 8 per group).

(L) Intratumoral antigen-specific $CD8^+$ T cells were analyzed on day 15 (n = 5 per group).

(M) Cells isolated from MC38-OT I tumor tissue were re-stimulated with SIINFEKL peptide, and GrmB, perforin, IFN-γ, and Ki67 were analyzed (n = 5 per group).

(N and O) TC-1-bearing mice were treated with E7 vaccine (3 doses indicated by black arrows in N) combined with GW6471 (10 mg/kg) or not. Tumor growth curve (N) and survival curve (O) were shown (n = 8 per group).

*$p < 0.05$; **$p < 0.01$; ***$p < 0.001$; ****$p < 0.0001$. (M) was analyzed with 2-way ANOVA. (L) was analyzed with 1-way ANOVA. Other data were analyzed with 2-tailed t test. The error bars represent SEMs. The error bars of (D) represent SDs. Each dot represents an individual mouse in (A), (B), (D)–(F), (L), and (M).

CellPress
OPEN ACCESS

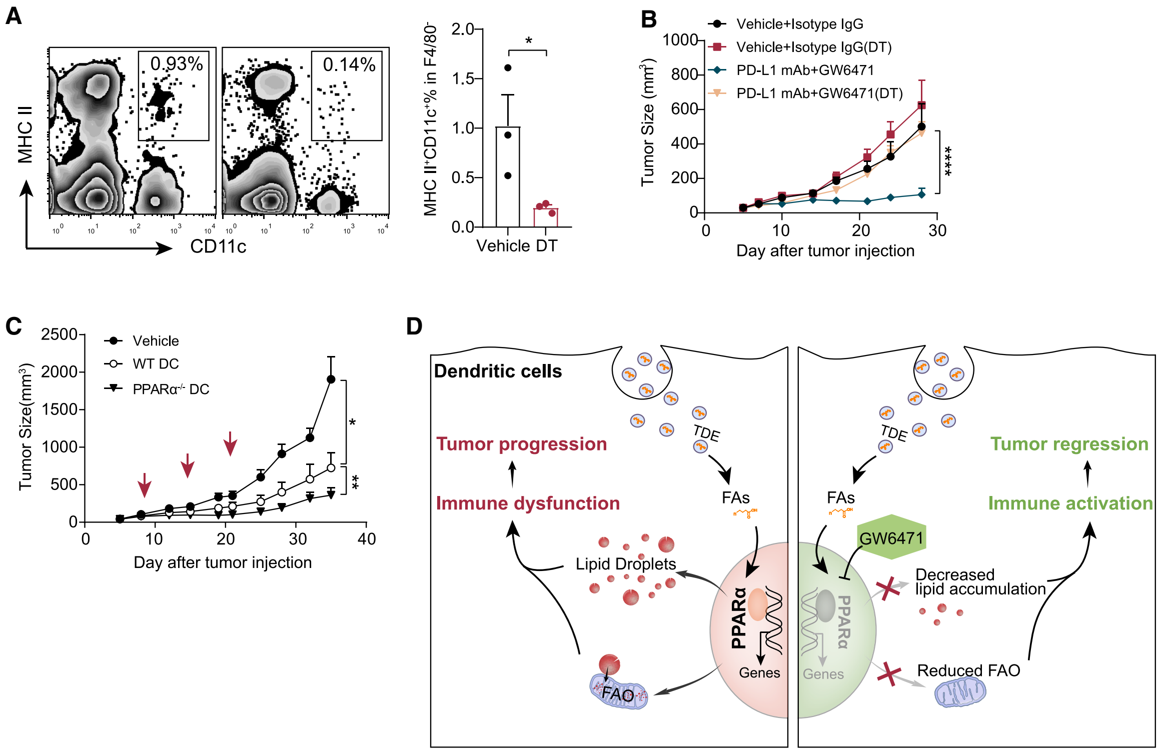

Figure 7. PPARα in DCs Is Required for Anti-tumor Immunity

(A and B) MC38-OT I tumors were subcutaneously (s.c.) injected in CD11c-DTR chimeric mice and treated with the combination of PD-L1 mAb (200 μg/dose/mouse) and GW6471 (10 mg/kg). Diphtheria toxin (DT) was intraperitoneally (i.p.) injected every other day before antibody therapy. The percentage of $CD45^{+}MHCII^{+}CD11c^{+}F4/80^{-}$ cells was measured (A). Each dot represents an individual mouse. The tumor growth curve was shown (B) (n = 10 per group).

(C) MC38-OT I-bearing mice were treated with WT DC or PPARα$^{-/-}$ DC (5×10^{6} per dose) s.c. 3 times (days 7, 14, and 21). The WT or PPARα$^{-/-}$ DC was pre-immunized with 10 μg/mLOVA$_{250\text{-}264}$ overnight. The tumor growth curve was shown (C) (n = 9 per group).

(D) Scheme of TDE-mediated DC immune dysfunction.

*$p < 0.05$; **$p < 0.01$; ****$p < 0.0001$. Data were analyzed with 2-tailed t test. The error bars represent SEMs.

to use PPARα$^{-/-}$ DCs as a vaccine to treat the tumor. We found that the PPARα$^{-/-}$ DC vaccine offered an improved anti-tumor effect compared with WT DC vaccine in the MC38-OT I tumor model (Figure 7C). Thus, our data collectively demonstrate TDEs, through activating PPARα signaling, conferring DC immune dysfunction. Blocking PPARα signaling with specific inhibitors restores the function of DCs and improves anti-tumor immunotherapy (Figure 7D). Importantly, these findings suggest that targeting PPARα may reverse the immune dysfunctional state of DCs and therefore could be a therapeutic strategy to improve anti-tumor immunotherapy.

DISCUSSION

In the TME, TIDCs have been viewed as immunologically dysfunctional due to their impaired capacity to present tumor-associated antigen and induce T cell proliferation (Gardner and Ruffell, 2016; Scarlett et al., 2012). Previous studies have suggested that abnormal lipid accumulation (Herber et al., 2010; Ramakrishnan et al., 2014) and metabolic reprogramming to FAO (Dong and Bullock, 2014; Loftus and Finlay, 2016; Sim et al., 2016) are frequently associated with functional defects of DC. This study reveals that TDEs induce DC immune dysfunction by transferring excessive FAs into DC, leading to lipid accumulation and enhanced FAO activity.

Exosomes are an important component in the TME and play a key role in tumor-host crosstalk. They can shuttle bioactive molecules from one cell to another or between different cell types, which fuels the metabolic activity of the recipient cells and therefore leads to extensive metabolic rewiring (Milane et al., 2015). Interestingly, TDEs are highly enriched in FAs compared with NEs, and we found that TDEs (Figure 3C) critically contribute to lipid accumulation in BMDCs. It is possible that, as tumor cells outcompete TIDCs for the availability of glucose in the TME, TIDCs turn to lipids as an alternative energy source, which may be provided by TDEs, the major FA carrier. Due to the limitation of the TDE isolation method used, however, our study does not exclude additional factors such as other microvesicles and soluble proteins in the TME that may cooperate with TDEs to induce DC dysfunction. Tumor cells use a variety of signaling molecules to communicate with DCs (DeVito et al., 2019). For example, tumor cells use paracrine Wnt5a/β-catenin signaling to activate PPARγ in DCs, leading to enhanced FAO and DC dysfunction (Holtzhausen et al., 2015; Zhao et al., 2018). Our present study,

together with previous findings, illustrate a complex communicating network that tumor cells use to reprogram local DCs for immune evasion.

In our study, we uncover that the amount of TDEs positively correlates with the degree of DC immune dysfunction in a dose-dependent manner due to the high level of FA content (Figures 2D and 2E). In fact, Herber et al. (2010) have reported that tumoral DCs can uptake FAs directly from tumor explant supernatant by Msr1 to induce lipid-laden DCs. However, our transcriptional analysis showed that TDEs did not increase the expression of Msr1 or CD36 in BMDCs, and that anti-Msr1 and anti-CD36 antibodies did not inhibit the uptake of TDEs by DCs (Figures S3D and S3E), suggesting that Msr1 and CD36 were not involved in TDE-induced lipid accumulation. Veglia et al. (2017) have reported that DCs in tumor-bearing hosts or tumor explant supernatant-treated BMDCs are defective in the cross-presentation of long antigen peptide (OVA), but not in the direct presentation of short antigen peptide ($OVA_{257-264}$). Interestingly, we observed that TDEs interfere with both direct presentation ($OVA_{257-264}$) (Figure S2C) and cross-presentation (OVA or $OVA_{250-264}$) of BMDCs (Figures 2C and S2C), suggesting that both TDEs and FAs in the TME may contribute to the lipid accumulation in DCs; however, they influence DC antigen presentation via distinct mechanisms.

High levels of exosome-containing FAs have been suggested to have diverse physiological effects (Haraszti et al., 2016). Mechanistically, our data show that LCFAs in TDEs activate PPARα in DCs, facilitating their switch toward FA catabolism and consequent inhibition of their function. As a master metabolic regulator, PPARα regulates FAO during fasting (König et al., 2009) and is also involved in immune cell functions. Previously, studies have reported that PPARα deficiency also decreases Tregs (Lei et al., 2010) and increases T lymphocyte proliferation (Zhang et al., 2015). In our study, we demonstrate that excess FAs carried by TDEs can be metabolized to produce energy by upregulating mitochondrial FAO via PPARα. However, energy production through mitochondria and FAO rather than glycolysis may lead to elevated reactive oxygen species (ROS) production. This increased production of ROS and subsequent peroxidation produces by-products such as 4-HNE, which triggers endoplasmic reticulum (ER) stress and blocks antigen cross-presentation (Cubillos-Ruiz et al., 2015; Ramakrishnan et al., 2014). In addition, PPARδ, another important member of the PPARs, has been reported to drive DCs toward a phenotype with reduced stimulatory effects on T cells (Jakobsen et al., 2006). However, our study shows that TDEs do not activate PPARβ/δ signaling in DCs.

Given the fact that macrophages also uptake large amounts of TDEs in the TME, we used an anti-CSF1R antibody to deplete macrophages and found that the combination therapy of an anti-PD-L1 antibody and GW6471 remains effective (Figures S6B and S6C). TAM *ex vivo* functional assays showed that GW6471 could not affect the ability of TAMs to prime T cells (Figures S6D and S6E). Moreover, although TDEs also induced BMDM lipid accumulation (Figure S6F), TDEs failed to suppress the immune function of BMDMs (Figure S6G). In sharp contrast, we demonstrate that DCs are necessary for the efficacy of the combination therapy using CD11c-DTR mice. Moreover, the anti-tumor effect of PPAR$\alpha^{-/-}$ DC vaccine further suggests the crucial role of PPARα in DC (Figure 7C). Studies using a DC-specific PPARα KO model are warranted to further clarify whether PPARα signaling within the DC population is required to regulate DC immune functions.

In summary, our studies reveal that TDEs, as carriers of FAs, directly increase cytoplasmic lipid levels and turn on the metabolic switch PPARα to induce LD accumulation and FAO, and finally suppress the T cell priming function of TIDCs. In addition, our results provide a promising immunotherapy combination strategy to maximize the induction, expansion, and cytotoxicity of tumor-specific $CD8^+$ T cells by restoring the function of TIDCs. As such, targeting PPARα can be exploited to improve DC-based cancer therapy.

STAR★METHODS

Detailed methods are provided in the online version of this paper and include the following:

- KEY RESOURCES TABLE
- RESOURCE AVAILABILITY
  - Lead Contact
  - Materials Availability
  - Data and Code Availability
- EXPERIMENTAL MODEL AND SUJECT DETAILS
  - Animals
  - Cell lines
- METHOD DETAILS
  - Generation of BMDCs or BMDMs
  - Preparation of FA Mix
  - Cell isolation from tissues
  - Exosome purification and characterization
  - Flow cytometry and antibodies
  - Lentiviral Transduction
  - T cell proliferation assays
  - Generation of Treg
  - IFN-γ ELISPOT and ELISA
  - Tumor Models
  - Bone marrow chimeras and dendritic cell depletion
  - RNA-Seq and bioinformatics analysis
  - Cellular energy metabolism analysis
  - Lipidomics studies
- QUANTIFICATION AND STATISTICAL ANALYSIS
  - Statistical Analysis

SUPPLEMENTAL INFORMATION

Supplemental Information can be found online at https://doi.org/10.1016/j.celrep.2020.108278.

ACKNOWLEDGMENTS

We thank Prof. Yangxin Fu, Prof. Hua Peng, and Dr. Zhichen Sun for kindly providing CD11c-DTR transgenic mice and constructive suggestions for our study. We thank Prof. Fuquan Yang for the lipidomic and proteomics analysis. We thank Prof. TaoTao Wei and Dr. ChaoJu Hua for the cellular energy metabolism analysis. We also thank Dr. Junying Jia for the flow cytometry analysis and cell sorting. This work is supported in part by the National Natural Science Foundation of China (81702823 to W.Z., and 81773288

to F.Z.); the National Health Commission for Significant New Drugs Development (2018ZX09201001-001-004 to C.Z.); NIH/NCI R37 CA249305 (to L.J.); DOD LCRP Career Development Award W81XWH20-1-0309 (to L.J.); the 2019 Breast Cancer Research Foundation-AACR Career Development Award for Translational Breast Cancer Research 19-20-26-JIN (to L.J.); the Elsa U. Pardee Foundation pilot grant (to L.J.); the Lung Cancer Research Foundation pilot grant (to L.J.); and UF Health Cancer Center pilot grant (to L.J.).

AUTHOR CONTRIBUTIONS

X.Y., W.Z., L.J., and W.L. designed the experiments. X.Y., W.Z., and L.J. wrote the manuscript. X.Y. and W.Z. performed the experiments and analyzed the data. X.Y., W.Z., and B.W. conducted the mass spectra experiments. Z.W., L.W., L.W., and H.T. contributed to the animal experiments. Y.J., R.C., X.W., Y.Q., F.Z., and C.Z. provided helpful advice.

DECLARATION OF INTERESTS

The authors declare no competing interests.

Received: September 25, 2019
Revised: June 14, 2020
Accepted: September 23, 2020
Published: October 20, 2020

REFERENCES

Apetoh, L., Locher, C., Ghiringhelli, F., Kroemer, G., and Zitvogel, L. (2011). Harnessing dendritic cells in cancer. Semin. Immunol. *23*, 42–49.

Cabodevilla, A.G., Sánchez-Caballero, L., Nintou, E., Boiadjieva, V.G., Picatoste, F., Gubern, A., and Claro, E. (2013). Cell survival during complete nutrient deprivation depends on lipid droplet-fueled β-oxidation of fatty acids. J. Biol. Chem. *288*, 27777–27788.

Calder, P.C. (2010). Lipid-laden dendritic cells fail to function. Cell Res. *20*, 1089–1091.

Cubillos-Ruiz, J.R., Silberman, P.C., Rutkowski, M.R., Chopra, S., Perales-Puchalt, A., Song, M., Zhang, S., Bettigole, S.E., Gupta, D., Holcomb, K., et al. (2015). ER Stress Sensor XBP1 Controls Anti-tumor Immunity by Disrupting Dendritic Cell Homeostasis. Cell *161*, 1527–1538.

Dáňová, K., Klapetková, A., Kayserová, J., Šedivá, A., Špíšek, R., and Jelínková, L.P. (2015). NF-κB, p38 MAPK, ERK1/2, mTOR, STAT3 and increased glycolysis regulate stability of paricalcitol/dexamethasone-generated tolerogenic dendritic cells in the inflammatory environment. Oncotarget *6*, 14123–14138.

DeVito, N.C., Plebanek, M.P., Theivanthiran, B., and Hanks, B.A. (2019). Role of Tumor-Mediated Dendritic Cell Tolerization in Immune Evasion. Front. Immunol. *10*, 2876.

Diamond, M.S., Kinder, M., Matsushita, H., Mashayekhi, M., Dunn, G.P., Archambault, J.M., Lee, H., Arthur, C.D., White, J.M., Kalinke, U., et al. (2011). Type I interferon is selectively required by dendritic cells for immune rejection of tumors. J. Exp. Med. *208*, 1989–2003.

Dong, H., and Bullock, T.N. (2014). Metabolic influences that regulate dendritic cell function in tumors. Front. Immunol. *5*, 24.

Everts, B., and Pearce, E.J. (2014). Metabolic control of dendritic cell activation and function: recent advances and clinical implications. Front. Immunol. *5*, 203.

Ferreira, G.B., Vanherwegen, A.S., Eelen, G., Gutiérrez, A.C.F., Van Lommel, L., Marchal, K., Verlinden, L., Verstuyf, A., Nogueira, T., Georgiadou, M., et al. (2015). Vitamin D3 Induces Tolerance in Human Dendritic Cells by Activation of Intracellular Metabolic Pathways. Cell Rep. *10*, 711–725.

Fonseca, P., Vardaki, I., Occhionero, A., and Panaretakis, T. (2016). Metabolic and Signaling Functions of Cancer Cell-Derived Extracellular Vesicles. Int. Rev. Cell Mol. Biol. *326*, 175–199.

Gardner, A., and Ruffell, B. (2016). Dendritic Cells and Cancer Immunity. Trends Immunol. *37*, 855–865.

Garris, C.S., Arlauckas, S.P., Kohler, R.H., Trefny, M.P., Garren, S., Piot, C., Engblom, C., Pfirschke, C., Siwicki, M., Gungabeesoon, J., et al. (2018). Successful Anti-PD-1 Cancer Immunotherapy Requires T Cell-Dendritic Cell Crosstalk Involving the Cytokines IFN-gamma and IL-12. Immunity *49*, 1148–1161.e7.

Grevengoed, T.J., Martin, S.A., Katunga, L., Cooper, D.E., Anderson, E.J., Murphy, R.C., and Coleman, R.A. (2015). Acyl-CoA synthetase 1 deficiency alters cardiolipin species and impairs mitochondrial function. J. Lipid Res. *56*, 1572–1582.

Haraszti, R.A., Didiot, M.C., Sapp, E., Leszyk, J., Shaffer, S.A., Rockwell, H.E., Gao, F., Narain, N.R., DiFiglia, M., Kiebish, M.A., et al. (2016). High-resolution proteomic and lipidomic analysis of exosomes and microvesicles from different cell sources. J. Extracell. Vesicles *5*, 32570.

Herber, D.L., Cao, W., Nefedova, Y., Novitskiy, S.V., Nagaraj, S., Tyurin, V.A., Corzo, A., Cho, H.I., Celis, E., Lennox, B., et al. (2010). Lipid accumulation and dendritic cell dysfunction in cancer. Nat. Med. *16*, 880–886.

Holtzhausen, A., Zhao, F., Evans, K.S., Tsutsui, M., Orabona, C., Tyler, D.S., and Hanks, B.A. (2015). Melanoma-Derived Wnt5a Promotes Local Dendritic-Cell Expression of IDO and Immunotolerance: Opportunities for Pharmacologic Enhancement of Immunotherapy. Cancer Immunol. Res. *3*, 1082–1095.

Jakobsen, M.A., Petersen, R.K., Kristiansen, K., Lange, M., and Lillevang, S.T. (2006). Peroxisome proliferator-activated receptor alpha, delta, gamma1 and gamma2 expressions are present in human monocyte-derived dendritic cells and modulate dendritic cell maturation by addition of subtype-specific ligands. Scand. J. Immunol. *63*, 330–337.

Kelly, D.P., and Scarpulla, R.C. (2004). Transcriptional regulatory circuits controlling mitochondrial biogenesis and function. Genes Dev. *18*, 357–368.

König, B., Rauer, C., Rosenbaum, S., Brandsch, C., Eder, K., and Stangl, G.I. (2009). Fasting Upregulates PPARalpha Target Genes in Brain and Influences Pituitary Hormone Expression in a PPARalpha Dependent Manner. PPAR Res. *2009*, 801609.

Lam, S.M., Wang, R., Miao, H., Li, B., and Shui, G. (2018). An integrated method for direct interrogation of sphingolipid homeostasis in the heart and brain tissues of mice through postnatal development up to reproductive senescence. Anal. Chim. Acta *1037*, 152–158.

Lei, J., Hasegawa, H., Matsumoto, T., and Yasukawa, M. (2010). Peroxisome proliferator-activated receptor α and γ agonists together with TGF-β convert human CD4+CD25- T cells into functional Foxp3+ regulatory T cells. J. Immunol. *185*, 7186–7198.

Liu, Z., Zhou, C., Qin, Y., Wang, Z., Wang, L., Wei, X., Zhou, Y., Li, Q., Zhou, H., Wang, W., et al. (2017). Coordinating antigen cytosolic delivery and danger signaling to program potent cross-priming by micelle-based nanovaccine. Cell Discov. *3*, 17007.

Loftus, R.M., and Finlay, D.K. (2016). Immunometabolism: Cellular Metabolism Turns Immune Regulator. J. Biol. Chem. *291*, 1–10.

Lu, J., Lam, S.M., Wan, Q., Shi, L., Huo, Y., Chen, L., Tang, X., Li, B., Wu, X., Peng, K., et al. (2019). High-Coverage Targeted Lipidomics Reveals Novel Serum Lipid Predictors and Lipid Pathway Dysregulation Antecedent to Type 2 Diabetes Onset in Normoglycemic Chinese Adults. Diabetes Care *42*, 2117–2126.

Malinarich, F., Duan, K., Hamid, R.A., Bijin, A., Lin, W.X., Poidinger, M., Fairhurst, A.M., and Connolly, J.E. (2015). High mitochondrial respiration and glycolytic capacity represent a metabolic phenotype of human tolerogenic dendritic cells. J. Immunol. *194*, 5174–5186.

Milane, L., Singh, A., Mattheolabakis, G., Suresh, M., and Amiji, M.M. (2015). Exosome mediated communication within the tumor microenvironment. J. Control. Release *219*, 278–294.

Mulcahy, L.A., Pink, R.C., and Carter, D.R. (2014). Routes and mechanisms of extracellular vesicle uptake. J. Extracell. Vesicles *3*. https://doi.org/10.3402/jev.v3.24641.

Palucka, K., and Banchereau, J. (2013). Dendritic-cell-based therapeutic cancer vaccines. Immunity *39*, 38–48.

Preynat-Seauve, O., Schuler, P., Contassot, E., Beermann, F., Huard, B., and French, L.E. (2006). Tumor-infiltrating dendritic cells are potent antigen-presenting cells able to activate T cells and mediate tumor rejection. J. Immunol. *176*, 61–67.

Ramakrishnan, R., Tyurin, V.A., Veglia, F., Condamine, T., Amoscato, A., Mohammadyani, D., Johnson, J.J., Zhang, L.M., Klein-Seetharaman, J., Celis, E., et al. (2014). Oxidized lipids block antigen cross-presentation by dendritic cells in cancer. J. Immunol. *192*, 2920–2931.

Rambold, A.S., Cohen, S., and Lippincott-Schwartz, J. (2015). Fatty acid trafficking in starved cells: regulation by lipid droplet lipolysis, autophagy, and mitochondrial fusion dynamics. Dev. Cell *32*, 678–692.

Rubinow, K.B., Wall, V.Z., Nelson, J., Mar, D., Bomsztyk, K., Askari, B., Lai, M.A., Smith, K.D., Han, M.S., Vivekanandan-Giri, A., et al. (2013). Acyl-CoA synthetase 1 is induced by Gram-negative bacteria and lipopolysaccharide and is required for phospholipid turnover in stimulated macrophages. J. Biol. Chem. *288*, 9957–9970.

Scarlett, U.K., Rutkowski, M.R., Rauwerdink, A.M., Fields, J., Escovar-Fadul, X., Baird, J., Cubillos-Ruiz, J.R., Jacobs, A.C., Gonzalez, J.L., Weaver, J., et al. (2012). Ovarian cancer progression is controlled by phenotypic changes in dendritic cells. J. Exp. Med. *209*, 495–506.

Sim, W.J., Ahl, P.J., and Connolly, J.E. (2016). Metabolism Is Central to Tolerogenic Dendritic Cell Function. Mediators Inflamm. *2016*, 2636701.

Spranger, S., Dai, D., Horton, B., and Gajewski, T.F. (2017). Tumor-Residing Batf3 Dendritic Cells Are Required for Effector T Cell Trafficking and Adoptive T Cell Therapy. Cancer Cell *31*, 711–723.e4.

Suvarna, K., Honda, K., Muroi, M., Kondoh, Y., Osada, H., and Watanabe, N. (2019). A small-molecule ligand of valosin-containing protein/p97 inhibits cancer cell-accelerated fibroblast migration. J. Biol. Chem. *294*, 2988–2996.

Svensson, K.J., Christianson, H.C., Wittrup, A., Bourseau-Guilmain, E., Lindqvist, E., Svensson, L.M., Mörgelin, M., and Belting, M. (2013). Exosome uptake depends on ERK1/2-heat shock protein 27 signaling and lipid Raft-mediated endocytosis negatively regulated by caveolin-1. J. Biol. Chem. *288*, 17713–17724.

Veglia, F., Tyurin, V.A., Mohammadyani, D., Blasi, M., Duperret, E.K., Donthireddy, L., Hashimoto, A., Kapralov, A., Amoscato, A., Angelini, R., et al. (2017). Lipid bodies containing oxidatively truncated lipids block antigen cross-presentation by dendritic cells in cancer. Nat. Commun. *8*, 2122.

Wahli, W., and Michalik, L. (2012). PPARs at the crossroads of lipid signaling and inflammation. Trends Endocrinol. Metab. *23*, 351–363.

Wculek, S.K., Khouili, S.C., Priego, E., Heras-Murillo, I., and Sancho, D. (2019). Metabolic Control of Dendritic Cell Functions: Digesting Information. Front. Immunol. *10*, 775.

Whiteside, T.L. (2016). Tumor-Derived Exosomes and Their Role in Cancer Progression. Adv. Clin. Chem. *74*, 103–141.

Yan, S., Yang, X.F., Liu, H.L., Fu, N., Ouyang, Y., and Qing, K. (2015). Long-chain acyl-CoA synthetase in fatty acid metabolism involved in liver and other diseases: an update. World J. Gastroenterol. *21*, 3492–3498.

Yang, Y., Li, C.W., Chan, L.C., Wei, Y., Hsu, J.M., Xia, W., Cha, J.H., Hou, J., Hsu, J.L., Sun, L., and Hung, M.C. (2018). Exosomal PD-L1 harbors active defense function to suppress T cell killing of breast cancer cells and promote tumor growth. Cell Res. *28*, 862–864.

Yao, C.H., Wang, R., Wang, Y., Kung, C.P., Weber, J.D., and Patti, G.J. (2019). Mitochondrial fusion supports increased oxidative phosphorylation during cell proliferation. eLife *8*, e41351.

Yi, L., He, J., Liang, Y., Yuan, D., Gao, H., and Zhou, H. (2007). Simultaneously quantitative measurement of comprehensive profiles of esterified and non-esterified fatty acid in plasma of type 2 diabetic patients. Chem. Phys. Lipids *150*, 204–216.

Zhang, M.A., Ahn, J.J., Zhao, F.L., Selvanantham, T., Mallevaey, T., Stock, N., Correa, L., Clark, R., Spaner, D., and Dunn, S.E. (2015). Antagonizing Peroxisome Proliferator-Activated Receptor α Activity Selectively Enhances Th1 Immunity in Male Mice. J. Immunol. *195*, 5189–5202.

Zhao, F., Xiao, C., Evans, K.S., Theivanthiran, T., DeVito, N., Holtzhausen, A., Liu, J., Liu, X., Boczkowski, D., Nair, S., et al. (2018). Paracrine Wnt5a-beta-Catenin Signaling Triggers a Metabolic Program that Drives Dendritic Cell Tolerization. Immunity *48*, 147–160.e7.

Zúñiga, J., Cancino, M., Medina, F., Varela, P., Vargas, R., Tapia, G., Videla, L.A., and Fernández, V. (2011). N-3 PUFA supplementation triggers PPAR-α activation and PPAR-α/NF-κB interaction: anti-inflammatory implications in liver ischemia-reperfusion injury. PLOS ONE *6*, e28502.

STAR★METHODS

KEY RESOURCES TABLE

REAGENT or RESOURCE	SOURCE	IDENTIFIER
Antibodies		
InVivoMab anti-mouse PD-L1 (B7-H1)	BioXCell	Cat # BE0101; RRID:AB_10949073
InVivoMab anti-mouse CSF1	BioXCell	Cat # BE0204; RRID:AB_10950309
InVivoMab rat IgG2a isotype control antibody	BioXCell	Cat # BE0089; RRID:AB_1107769
Anti-mouse CD8a APC	Biolegend	Cat # 100712; RRID:AB_312751
Anti-mouse CD8a PerCP/Cyanine5.5	Biolegend	Cat # 100733; RRID:AB_2075239
Anti-mouse CD4 APC	Biolegend	Cat # 100516; RRID:AB_312719
Anti-mouse CD274(PD-L1) PE	Biolegend	Cat # 124307; RRID:AB_2073557
Anti-mouse CD3 PE/Cy7	Biolegend	Cat # 100220; RRID:AB_173205
Anti-mouse CD11b PE	eBioscience	Cat # 12-0112-82; RRID:AB_2734869
Anti-mouse CD11c FITC	Biolegend	Cat # 117306; RRID:AB_313775
Anti-mouse CD11c APC/Cy7	Biolegend	Cat # 117323; RRID:AB_830646
Anti-mouse CD11c APC	Biolegend	Cat # 117310; RRID:AB_313779
Anti-mouse F4/80 BV785	Biolegend	Cat # 123141; RRID:AB_2563667
Anti-mouse Gr-1 BV605	Biolegend	Cat # 108441; RRID:AB_2562401
Anti-mouse IFN-γ PerCP/Cyanine5.5	eBioscience	Cat # 45-7311-82; RRID:AB_1107020
Anti-mouse Granzyme B FITC	eBioscience	Cat # 11-8898-82; RRID:AB_10733414
Anti-mouse Perforin PE	eBioscience	Cat # 12-9392-82; RRID:AB_466243
Anti-mouse Ki67 BV421	Biolegend	Cat # 151208; RRID:AB_2629748
Anti-mouse CD80 FITC	Biolegend	Cat # 104706; RRID:AB_313127
Anti-mouse CD86 PE	Biolegend	Cat # 105007; RRID:AB_313150
Anti-mouse CD40 PE	eBioscience	Cat # MA5-17855; RRID:AB_2539239
Anti-mouse OX40L PE	Biolegend	Cat # 108805; RRID:AB_313404
Anti-mouse MHC II PE/Cy7	Biolegend	Cat # 107630; RRID:AB_2069376
Anti-mouse MHC II APC	Biolegend	Cat # 107614; RRID:AB_313329
Anti-Mouse OVA257-264 (SIINFEKL) peptide bound to H-2Kb PE	Biolegend	Cat # 141603; RRID:AB_10897938
Anti-mouse CD45 BV605	Biolegend	Cat # 103139; RRID:AB_2562341
Anti-mouse CD45 PerCP/Cyanine5.5	Biolegend	Cat # 103132; RRID:AB_893340
Anti-mouse Ly6C PerCP/Cyanine5.5	Biolegend	Cat # 128012; RRID:AB_1659241
Mouse SR-AI/MSR1 Affinity Purified Ab	R&D	Cat # AF1797; RRID:AB_2148246
Chemicals, Peptides, and Recombinant Proteins		
BODIPY 493/503	Invitrogen	Cat # D3922
iTAg Tetramer/APC - H-2 Kb OVA (SIINFEKL)	MBL	Cat # TB-5001-2
Carbonyl cyanide 4-(trifluoromethoxy) phenylhydrazone (FCCP)	Sigma-Aldrich	Cat # C2920
Bovine Serum Albumin, fatty acid free	Sigma-Aldrich	Cat # A8806
Polybrene	Sigma-Aldrich	Cat # TR-1003
Ovalbumin from Egg White	BBI	Cat # A003056-0100
PHRODO GREEN STP ESTER	invitrogen	Cat # P35369
HCS LIPIDTOX DEEP RED NEUTRAL LIPID STAIN	invitrogen	Cat # H34477
PKH67 Green Fluorescent Cell Linker Midi Kit	Sigma	Cat # MIDI67-1KT
Collagenase IV	Life	Cat # 17104019-1
CFSE	eBioscience	Cat # 65-0850-84
CELL-TAK CELL TISSUE ADHESIVE	Biocoat	Cat # 354240

(*Continued on next page*)

Continued

REAGENT or RESOURCE	SOURCE	IDENTIFIER
Etomoxir sodium salt hydrate	Sigma	Cat # E1905-25MG
GW6471	R&D	Cat # 4618/50
GW4869	Selleck	Cat # S7609-5mg
Diphtheria Toxin	Merck Millipore	Cat # 322326-1MG
Triacsin C	Sigma	Cat # T4540-1MG
C75	Sigma	Cat # C5490-5MG
PMA	Sigma	Cat # P1585-1MG
Ionomycin	Sigma	Cat # I3909-1ML
5-tetradecyl-oxy-2-furoic acid	Sigma	Cat # T6575-25MG
Arachidonic acid	Sigma	Cat # A3611-10MG
Oleic acid	Sigma	Cat # O1008-1G
Palmitic acid	Sigma	Cat # 27734-1KG
Stearic acid	Merck Millipore	Cat # 569398-25MG
Recombinant Murine IL-2	Peprotech	Cst # 212-12
Recombinant Murine GM-CSF	Peprotech	Cat # 315-03
Recombinant Murine M-CSF	Peprotech	Cat # 212-12
Critical Commercial Assays		
MojoSort Mouse CD8 T cell Isolation Kit	Biolegend	Cat # 480035
MojoSort Mouse CD4 T cell Isolation Kit	Biolegend	Cat # 480005
10* Permeabilization buffer	eBioscience	Cat # 00-8333-56
Fix/Permeabilization buffer set	eBioscience	Cat # 88-8824-00
eBioscience Brefeldin A Solution (1000X)	eBioscience	Cat # 00-4506-51
10*RBC Lysis Buffer	eBioscience	Cat # 00-4300-54
ExoAb Antibody Kit	SBI	Cat # EXOAB-KIT-1
ExoQuick TC	SBI	Cat # EXOTC50A-1
Lactate Colorimetric/Fluorometric Assay Kit	Biovision	Cat # 93-K607-100
Mouse IFN-γ ELISA MAX Deluxe	Biolegend	Cat # 430805
IFN-γ ELISPOT assay kit	BD	Cat # 552569
Mouse TGF-beta 1 DuoSet ELISA	R&D	Cat # DY1679-05
BCA Protein Assay Kit	Pierce	Cat # 23225
Fast SYBR Green Master Mix	Applied Biosystems	Cat # 4385612
Seahorse XF24 Fluxpak mini	Agilent	Cat # 100867-100
Experimental Models: Cell lines		
DC2.4 lines	Laboratory of Mingzhao Zhu	N/A
TC1 lines	ATCC	Cat # JHU-1
4T1 lines	ATCC	Cat # CRL-2539
B16/F10 lines	ATCC	Cat # CRL-6475
MC38 lines	Laboratory of Yangxin Fu	N/A
MC38-OT I lines	Laboratory of Yangxin Fu	N/A
293T lines	Laboratory of Guangxia Gao	N/A
Experimental Models: Organisms/Strains		
C57BL/6	Vital River Laboratory Animal Technology	Cat # 213
BABL/c	Vital River Laboratory Animal Technology	Cat # 211
C57BL/6-Tg (TcraTcrb)1100Mjb/J	The Jackson Laboratory	Cat # N000208
PPARα Knockout mice	Cyagen Biosciences	Cat # KOCMP-21034-Ppara
GPF-Foxp3 mice	Laboratory of Yangxin Fu	N/A

(*Continued on next page*)

Continued

REAGENT or RESOURCE	SOURCE	IDENTIFIER
CD11c-DTR mice	Laboratory of Yangxin Fu	N/A
Recombinant DNA		
pCT-CD9-GFP (pCMV, Exosome/Secretory, CD9 Tetraspanin Tag)	SBI	Cat # CYTO122-PA-1
psPAX2	Addgene	Cat # Addgene plasmid#12260
pMD2.G	Addgene	Cat # Addgene plasmid#12259
Oligonucleotides		
PPARα-F: AACATCGAGTGTCGAATATGTGG	This paper	N/A
PPARα-R: CCGAATAGTTCGCCGAAAGAA	This paper	N/A
18S-F: CGGCTACCACATCCAAGGAA	This paper	N/A
18S-R: GCTGGAATTACCGCGGCT	This paper	N/A
Deposited Data		
RNA-Seq (generated)	This paper	GEO: GSE155881
Software and Algorithms		
GraphPad Prism 8	Graphpad Software	https://www.graphpad.com:443; RRID:SCR_002798
FlowJo	BD	https://www.flowjo.com/solutions/flowjo; RRID:SCR_008520
R Project	R	http://www.r-project.org/; RRID:SCR_001905
ImageJ	ImageJ public freeware	https://imagej.nih.gov/nih-image/index.html; RRID:SCR_003073

RESOURCE AVAILABILITY

Lead Contact

Further information and requests for resources and reagents should be directed to and will be fulfilled by the Lead Contact, Wei Liang (weixx@sun5.ibp.ac.cn).

Materials Availability

This study did not generate new unique reagents.

Data and Code Availability

The accession number for the RNA-seq data reported in this paper is GEO: GSE155881.

EXPERIMENTAL MODEL AND SUJECT DETAILS

Animals

Female BALB/c or C57BL/6 mice (6-8 weeks old) were purchased from Vital River Laboratory Animal Technology (Beijing, China). OT-I T cell receptor-transgenic mice (C57BL/6-Tg (TcraTcrb)1100mjb) whose T cell receptors recognize ovalbumin (OVA) residues 257-264 in the context of $H2K^b$ were obtained from the Jackson Laboratory (Bar Harbor, ME, USA). CD11c-DTR mice and GFP-Foxp3 mice were provided by Prof. Yangxin Fu (University of Texas, Southwestern Medical Center, Texas, USA). PPARα knockout mice were obtained from Cyagen Biosciences (China). All animal experiments were performed according to the institutional ethical guidelines on animal care and the protocols used for this study were approved by the Animal Care and Use Committee at the Institute of Biophysics, Chinese Academy of Sciences.

Cell lines

Murine breast cancer 4T1 (on BALB/c mice), cervical carcinoma TC-1 (on C57BL/6 mice), colon carcinoma MC38-OT I (harboring ovalbumin 257-264 (referred to as OT-I peptide) antigen), MC38 and melanoma B16/F10 (on C57BL/6 mice) were cultured in 5% CO_2 and maintained in RPMI 1640 or DMEM medium supplemented with 10% FBS (BI, Isreal) 100 U/ml penicillin, and 100 μg/ml streptomycin. 4T1, TC-1 and B16/F10 were obtained from ATCC, MC38 and MC38-OT I were obtained from the laboratory of Yangxin Fu, and the test for mycoplasma infection were negative.

METHOD DETAILS

Generation of BMDCs or BMDMs

BMDCs or BMDMs were prepared from the femurs of C57BL/6 mice at 8-10 weeks of age and were cultured for 7 days with two replenishments of medium without disturbing the cells. BMDCs were cultured in RPMI 1640 medium with 10% FBS, 0.1% β-mercaptoethanol and 20 ng/ml rmGM-CSF. BMDMs were cultured in RPMI 1640 medium with 10% FBS, 0.1% β-mercaptoethanol and 20 ng/ml rmM-CSF.

Preparation of FA Mix

PA (C16:0), SA (C18:0), OA (C18:1) and AA (C20:4n6) were prepared in 96% ethanol with 200 mM stock solutions by heating and constant shaking at 70°C and 37°C, respectively. Then fatty acids (FAs) were diluted 1:10 in prewarmed 10% fatty acid free BSA receiving a final concentration of 20 mM. Immediately, FA-BSA mixture was vortexed for 30 s and incubated while gently shaking for 15 min at 55°C and 37°C. After filtering through a 0.2 μm filter under sterile conditions, the opaque solution obtained limpid appearance again and was stored at −20°C for two months.

Cell isolation from tissues

Tumor tissues were collected, minced into small pieces, and digested in 2 mg/ml collagenase Type IV at 37°C for 1 hour. The digested tumor tissues were then filtered through a 70 μm cell strainer to make a single-cell suspension. DCs or macrophages from tumor were sorted by FACS Aria (BD, San Jose, CA, USA).

Exosome purification and characterization

Tumor cells were cultured in RPMI 1640 media supplemented with 10% exosome-depleted FBS (BI, Israel). Supernatant of tumor cells culture was collected 48 hours after cell reached 80% confluence. Then the supernatant was centrifuged at 4000 rpm for 2 hours to remove cell debris, followed by 4000 rpm centrifuge for 30 min using 100 KDa MWCO to make the exosome-concentrated solution. The exosome was isolated by exosome quick extraction solution. The protein content of exosome was determined by BCA protein assay kit. The characterization of exosomes was confirmed by measuring expression of exosome-specific markers ALIX, HSP70, TSG101, CD81 and CD9 (SBI) by western blot analysis and Transmission Electron Microscopy (FEI spirit 120kV, USA). Exosomes were labeled with PKH67 membrane dye at 37°C for 5 minutes, and analyzed by confocal microscope (FV1000, Olympus). All the unspecified exosomes used in this study were from either TC-1 or MC38 tumor cells.

Flow cytometry and antibodies

TIDCs, spleen DCs, BMDCs or other cells were stained for surface markers using CD11c-APC, CD45-BV605, MHC II-PE/Cy7, PE-CD11b, Ly6C-Percp/Cy5.5, F4/80-BV785 followed by staining with BODIPY 493/503 at 0.5 μg/ml in PBS or LipidTOX™ Deep Red neutral lipid stain for 15 minutes at room temperature in the dark. For intracellular staining, cells were stimulated with 100 ng/ml PMA and 0.5 μg/ml ionomycin at 37°C for 5 hours, adding Brefeldin A (10 mg/ml) to accumulate intracellular cytokines. All the experiments were performed on FACSCalibur and FACSAria IIIu (BD Biosciences, San Jose, CA, USA) and analyzed with FlowJo 7.6.1 software (BD).

Lentiviral Transduction

For exosome tracing studies, 293T cells were transfected with 5 μg of the plasmids (pCT-CD9-GFP (SBI):psPAX2:pMD2.G (Addgene) = 4:3:1) and with 7.5 μL Lipofectamine 3000 (Invitrogen) for the production of retrovirus. Then, TC-1 or MC38 cells were transduced with this lentiviral vector pCT-CD9-GFP. Transfection efficiency was analyzed by flow cytometry.

T cell proliferation assays

$CD8^+$ T cells were harvested from OT-I transgenic mice and purified by magnetic beads. 5×10^4 BMDCs or BMDMs were pulsed with 2 mg/ml endotoxin-free OVA in the presence or absence of exosomes with or without inhibitors, unless indicated otherwise. After 48 hours of incubation, BMDCs or BMDMs were washed off the antigen. 2×10^5 CFSE-labeled OT-I $CD8^+$T cells were incubated with the BMDCs (Ratio of DC: T = 1:8) or BMDM (Ratio of BMDM: T = 1:10). After 3 days co-incubation, proliferation of $CD8^+$ T cells were analyzed by double gating on CD8 and CFSE.

Generation of Treg

$CD4^+$ T cells were isolated from GFP-Foxp3 transgenic mice and purified by magnetic beads. 2×10^4 BMDCs were treated with or without exosomes in presence or absence of inhibitor for 48 hours. Then washed BMDCs were cultured with purified $CD4^+$ T cells (Ratio of DC: T = 1:10). On day 3, 100 U/ml IL-2 was added, and on day 7, percentage of Tregs ($CD4^+GFP^+$) were analyzed by flow cytometry.

IFN-γ ELISPOT and ELISA

Draining LNs from MC38-OT-I tumor-bearing mice were isolated and single-cell suspensions were prepared. 5×10^4 cells were assayed per well, stimulated with 20 μg/ml OT-I peptide (SIINFEKL) or not. 48 hours later, spots indicating IFN-γ-producing T cells were enumerated by ImmunoSpot Analyzer (CTL). IFN-γ in supernatants were detected by ELISA method.

Tumor Models

Tumor cells were subcutaneously injected at 2×10^4 cells per mouse (in 4T1 tumor model), at 5×10^4 cells per mouse (in TC-1 tumor model), at 1.2×10^5 cells per mouse (in B16/F10 tumor model), at 2.5 or 5×10^5 cells per mouse (in MC38 or MC38-OT-I tumor model). Mice were randomized to treatment groups when tumors reached certain sizes. Tumor volumes were measured twice a week and calculated as length × width × width/2. All animal experiments were performed according to the institutional ethical guidelines on animal care and the protocols used for this study were approved by the Animal Care and Use Committee at the Institute of Biophysics, Chinese Academy of Sciences.

Bone marrow chimeras and dendritic cell depletion

To generate bone marrow chimeras, WT C57BL/6 mice were sublethally irradiated at 10 Gy. 24 hours later, bone marrow cells in femurs of donor mice were harvested, washed, resuspended in PBS and i.v injection into the irradiated mice. For CD11c-DTR bone marrow chimera construction, 5×10^6 cells per mouse were injected. For depletion of DC, diphtheria toxin (500 ng/dose/mouse) was administered i.p. to CD11c-DTR chimeras every other day before treatment (Spranger et al., 2017).

RNA-Seq and bioinformatics analysis

To assess the level of gene expression, RNA was extracted from purified CTR, TDE-treated and NE-treated BMDCs with TRIzol reagent (Invitrogen). After the quality of the total RNA was verified with an Agilent 2100 Bioanalyzer, the samples were processed using Illumina Novaseq 6000 system. This system incorporates oligo(dT) and random primers for amplification at the 3′ end throughout the whole transcriptome. The RNA-Seq raw data were processed through the standard RNA-Seq analysis pipeline. Briefly, read alignment was examined using TopHat2 version 2.1.1 (http://ccb.jhu.edu/software/tophat/in). Differential-expression analysis was carried out with DESeq2 version 1.24.0 http://bioconductor.org/about/removed-packages/) in R v.3.3.1 (http://cran.r-project.org/). Genes were considered to be differentially expressed if the adjusted *P* was less than 0.05. Metabolic pathways were defined by MAJOBIO CLOUD (https://cloud.majorbio.com) after initial gene set comparison. The identified gene set involved in a specific metabolic pathway was further analyzed with GSEA (https://www.broadinstitute.org/gsea/index.jsp).

Cellular energy metabolism analysis

DC energy metabolism was measured using the XF^e24 extracellular flux analyzer (Agilent). In brief, 3×10^5 BMDCs per well were treated with or without exosomes for 48 hours prior to XF analysis. For standard OCR analysis, XF media (with 10mM glucose) was used to wash cells, a final concentration of 1 μM oligomycin, 1.5 μM FCCP, 100 nM rotenone and 1 μM of antimycin-A were injected through XF^e24 port A-C. To determine the rate of mitochondrial FAO, BMDCs were co-cultured with or without exosomes and GW6471 for 48 hours and subsequently seeded at equal densities in substrate-limited medium (DMEM with 0.5 mM glucose, 1 mM glutamine, 0.5 mM carnitine and 1% FBS) and incubated overnight. 45 minutes before the beginning of OCR measurement, the cells were changed into FAO Assay Medium (111 mM NaCl, 4.7 mM KCl, 2 mM $MgSO_4$, 1.2 mM Na_2HPO_4, 2.5 mM glucose, 0.5 mM carnitine and 5 mM HEPES). After the baseline OCR is stabilized in FAO Assay Medium, ETO (100 μM) was added to reveal the amount of FAO-associated OCR (subtracting post-ETO OCR from basic OCR). Lactate in supernatants was detected by Lactate Colorimetric/Fluorometric Assay Kit, according to the manufacturer's instructions.

Lipidomics studies

To compare the fatty acids in TDEs versus TDE-treated BMDCs, lipid extraction and methylation of fatty acids were performed as published by Yi,L previously (Yi et al., 2007). All GC–MS/MS analyses were performed by an Agilent 7890A series GC system coupled with an Agilent 7000B QqQMS (Agilent Technologies Inc., USA). A sample of 1.0 μL was injected, and the injection mode was splitless, the scan range was set at m/z 50–550 in the full scan mode. The library search and mass spectral matching were conducted using NIST11.L. Calculation of peak area was performed using Agilent Mass Hunter quantitative software.

To compare the fatty acids in TDEs and NEs, the lipids in exosomes were extracted using the improved Bligh/Dyer extraction method (Lu et al., 2019). Then the samples were reconstituted in the isotope mixed standards, followed by analysis on Exion UPLC-QTRAP 6500 Plus LC/MS (Sciex) in electrospray ionization (ESI) mode with the conditions optimized as follows: curtain gas = 20, ion spray voltage = 5500 V, temperature = 400°C, ion source gas 1 = 35, Ion source gas 2 = 35. Using Phenomenex Luna silica 3 μm (inner diameter 150x2.0mm) chromatography column to separate lipids. Mass spectrometry multiple reaction monitoring is used for various forms of lipid identification and quantitative analysis (Lam et al., 2018) (supported by Lipidall Technologies Company Limited).

QUANTIFICATION AND STATISTICAL ANALYSIS

Statistical Analysis

All statistical analysis was performed using GraphPad Prism 8 (GraphPad). The variations of data were evaluated as mean ± SEM or mean ± SD. The statistical significance of the differences between two groups was measured by the unpaired 2-tailed Student's t test, and one-way or two-way ANOVA were performed for multi-group comparisons. A value of $p < 0.05$ was considered statistically significant (*$p < 0.05$; **$p < 0.01$; ***$p < 0.001$; ****$p < 0.0001$).

Cell Reports, Volume *33*

Supplemental Information

PPARα Inhibition Overcomes

Tumor-Derived Exosomal Lipid-Induced

Dendritic Cell Dysfunction

Xiaozhe Yin, Wenfeng Zeng, Bowen Wu, Luoyang Wang, Zihao Wang, Hongjian Tian, Luyao Wang, Yunhan Jiang, Ryan Clay, Xiuli Wei, Yan Qin, Fayun Zhang, Chunling Zhang, Lingtao Jin, and Wei Liang

Figure S1

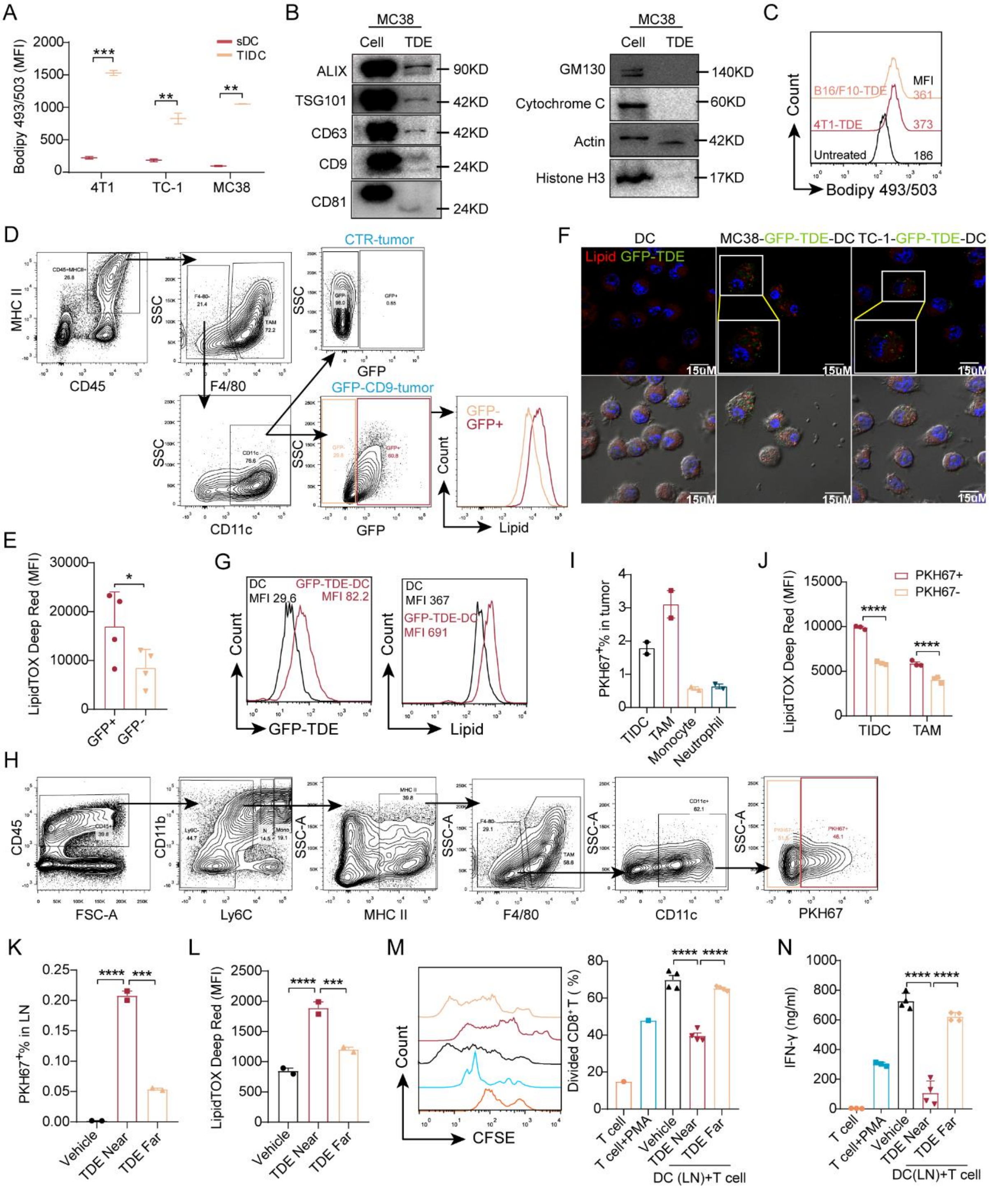

A
B
C
D
E
F
G
H
I
J
K
L
M
N
MC38
Cell
TDE
ALIX
TSG101
CD63
CD9
CD81
90KD
42KD
24KD
GM130
Cytochrome C
Actin
Histone H3
140KD
60KD
17KD
sDC
TIDC
Bodipy 493/503 (MFI)
4T1
TC-1
MFI
B16/F10-TDE
4T1-TDE
Untreated
361
373
186
Count
Bodipy 493/503
CTR-tumor
GFP-CD9-tumor
MHC II
CD45
SSC
F4/80
GFP
CD11c
GFP-
GFP+
Lipid
DC
MC38-GFP-TDE-DC
TC-1-GFP-TDE-DC
Lipid GFP-TDE
15uM
LipidTOX Deep Red (MFI)
GFP-TDE-DC
MFI 29.6
MFI 82.2
MFI 367
MFI 691
GFP-TDE
PKH67+% in tumor
TAM
Monocyte
Neutrophil
PKH67+
PKH67-
FSC-A
CD11b
Ly6C
SSC-A
PKH67
PKH67+% in LN
Vehicle
TDE Near
TDE Far
CFSE
Divided CD8+T (%)
T cell
T cell+PMA
DC (LN)+T cell
IFN-γ (ng/ml)
DC(LN)+T cell

Figure S1 (related to Figure 1). TDEs induce lipid laden DC

(A) Intracellular lipid level of $CD45^+MHC\ II^+CD11c^+\ F4/80^-$ DCs in spleen (sDC) and tumor (TIDC) from various tumor models was measured by Bodipy 493/503 staining (*n*=3 per group).

(B) Western blot reveals exosome markers in both cell lysate and TDE.

(C) Intracellular lipid levels in BMDCs treated with 4T1 or B16/F10 tumor cells derived exosomes (400 μg/ml) for 24 h. Representative of two independent experiments.

(D) Flow cytometry and gating strategy of tumor infiltrating DCs in GFP-CD9-tumor or CTR-tumor.

(E) Intracellular lipid levels in GFP^+ or GFP^- TAMs were analyzed by flow cytometry, related to Figure 1I-K. Each dot represents an individual mouse.

(F) Representative confocal images showing the intracellular lipid level of DCs treated with 100 μg/ml GFP-TDE for 8 h. Scale bar = 15 μm.

(G) GFP-TDE uptake and lipid level of DCs treated with 400 μg/ml GFP-TDE for 24 h.

(H) MC38 tumors (on day 10) were harvested 48 h after 1000 μg PKH67 labeled TDEs injection. Flow cytometry and gating strategy of tumor infiltrating myeloid cells.

(I and J) Relative proportions of tumor infiltrating myeloid cells as a percentage of total $PKH67^+$ cells (I). TIDC ($CD45^+Ly6C^-MHC\ II^+CD11c^+F4/80^-$), TAM ($CD45^+Ly6C^-MHC\ II^+F4/80^+$), Monocytes ($CD45^+CD11b^+Ly6C^{hi}$), Neutrophils ($CD45^+CD11b^+Ly6C^{mid}$). Lipid content in $PKH67^+$ or $PKH67^-$ TIDC and TAM were analyzed by flow cytometry (*n*=4) (J). Each dot represents an individual mouse.

(K-N) 1000 μg PKH67 labeled TDEs were injected into the footpad of wild type C57BL/6 mice and measured the phenotype change of DCs in popliteal lymph nodes (LN)(near) and inguinal lymph nodes (far) at 48 h post injection. Frequency of DC populations as a percentage of total $PKH67^+$ cells (K). Lipid content was analyzed by flow cytometry (L). The isolated DCs ($CD45^+Ly6C^-MHC\ II^+CD11c^+F4/80^-$) from lymph nodes were treated with 2 mg/ml OVA overnight, then co-cultured with OT I $CD8^+$ T cells for 3 days. Proliferation of $CD8^+$ T was analyzed by CFSE dilution (M) and the production of IFN-γ of $CD8^+$ T cells was analyzed by ELISA (N). T: undivided OT-I cells as a negative control. PMA: OT-I T cells primed by PMA and ionomycin as a qualification control. Each dot represents an individual mouse.

$^{**}P < 0.01$; $^{***}P < 0.001$; $^{****}P < 0.0001$. (A) and (J) were analyzed with two-way ANOVA. (E) was analyzed with 2-tailed *t* test. Other data were analyzed with one-way ANOVA. Error bars represent SEM. The error bars of (N) represents SD.

Figure S2

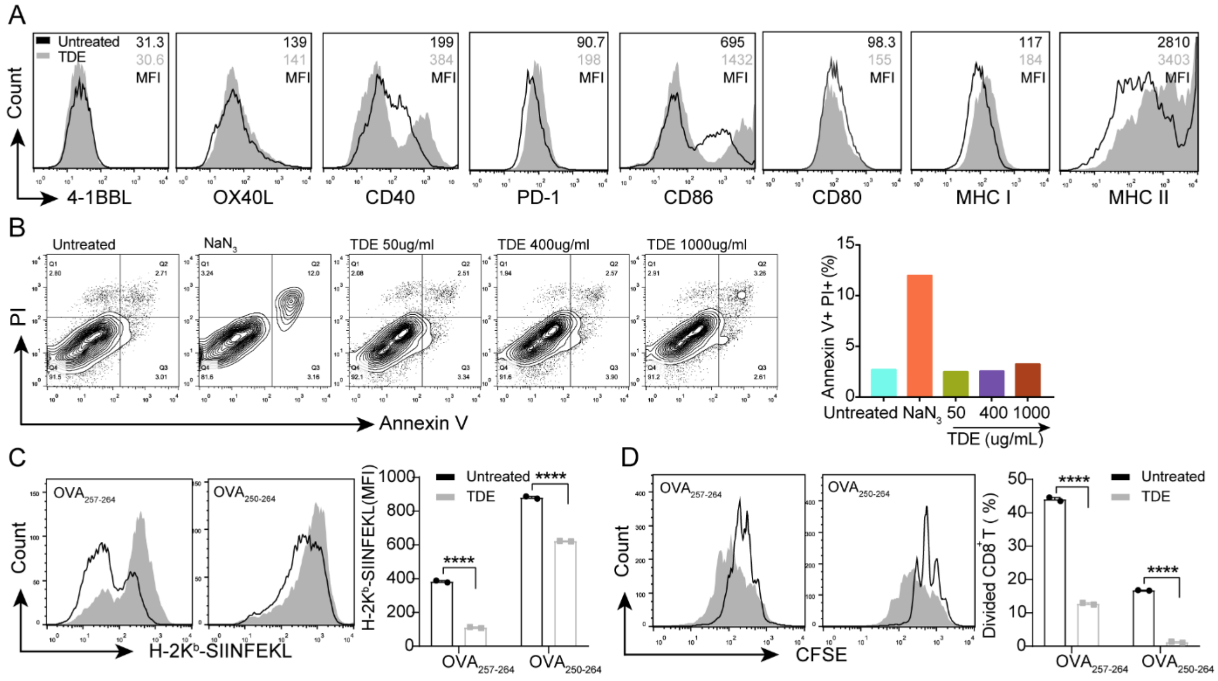

Figure S2 (related to Figure 2). TDEs interfere DC immune function

(A) BMDCs were treated with TDEs (400 μg/ml) for 24 h. The expression of surface markers for BMDCs maturation were analyzed. Representative of two independent experiments.

(B) Cytotoxicity analysis of TDEs in BMDCs with 24 h. Representative of two independent experiments.

(C) BMDCs were treated with 10 μg/ml OVA$_{257-264}$ or OVA$_{250-264}$ in the presence or absence of TDEs (200 μg/ml) for 24 h. Antigen presentation of BMDCs was assessed by staining with H-2Kb-SIINFEKL antibody. Representative of two independent experiments.

(D) BMDCs were treated with 10 μg/ml OVA$_{257-264}$ or OVA$_{250-264}$ in the presence or absence of TDEs (200 μg/ml) for 48 h, which were subsequently washed off the antigen and co-cultured with OT I CD8$^+$ T cells for 3 days. Proliferation of OT I CD8$^+$ T cells was analyzed by CFSE dilution. Representative of two independent experiments.

*****P* < 0.0001. Data were analyzed with two-way ANOVA. Error bars represent SEM.

Figure S3

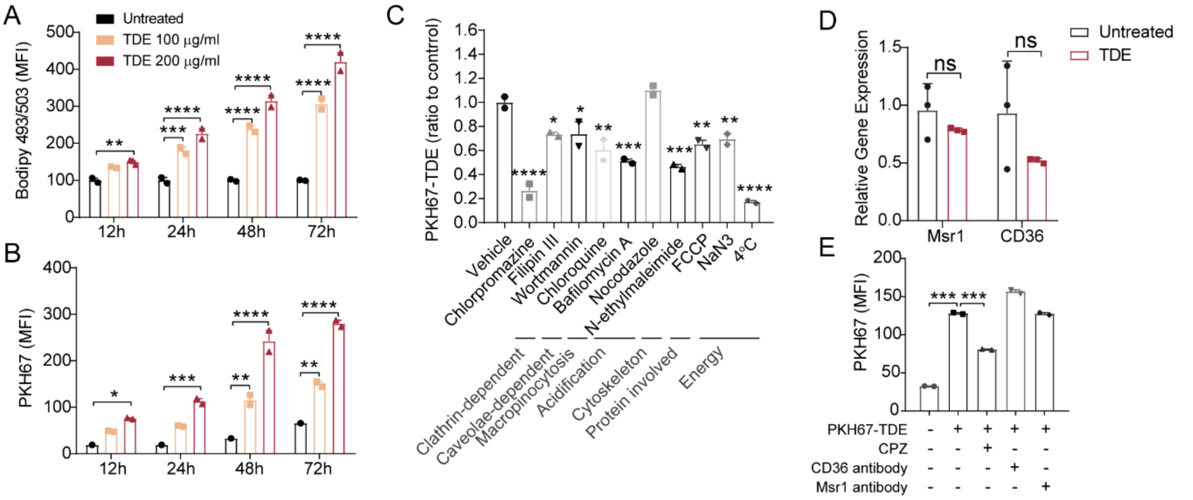

Figure S3 (related to Figure 3). The mechanism of TDEs uptake by DCs

(A and B) BMDCs were treated with 100 μg/ml or 200 μg/ml PKH67-labeled TDEs for different periods. 12, 24, 48 and 72 hours later, BMDCs were collected and the fluorescence intensity of Bodipy 493/503 (A) and PKH67 (B) were analyzed by flow cytometry respectively.

(C) Effects of endocytic inhibitors on internalization of PKH67-labeled TDEs. BMDCs were pre-treated with various inhibitors for different lengths of time (see Table 1), and then incubated with 100 μg/ml PKH67-labeled exosomes for 8 h. Internalized exosomes were detected by flow cytometry. Representative of two independent experiments.

(D) Expression of the indicated transcripts were assessed by RNA-Seq.

(E) Flow cytometry analyzed the effect of CD36 antibody or Msr1 antibody on internalization of PKH67 labeled TDEs. BMDCs were pre-treated with CD36 antibody or Msr1 antibody for 15 min and then incubated with 50 μg/ml PKH67 labeled TDEs for 8 h. CPZ, Chlorpromazine.

*$P < 0.05$; **$P < 0.01$; ***$P < 0.001$; ****$P < 0.0001$; ns, not significant. (C) and (E) were analyzed with one-way ANOVA. Other data were analyzed with two-way ANOVA. Error bars represent SEM.

Figure S4

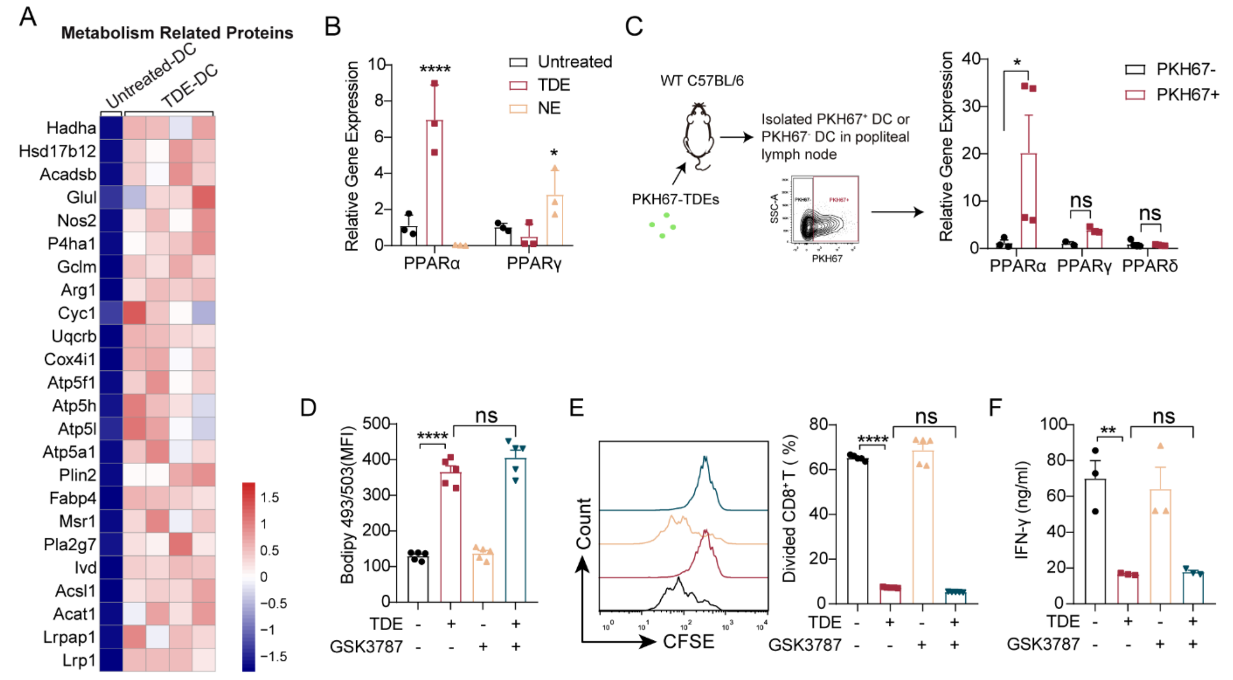

Figure S4 (related to Figure 4). Proteomics analysis of BMDC

(A) Upregulation of genes involved in the metabolism of BMDCs cultured with or without TDEs were analyzed by proteomics.

(B) Expression of the indicated transcripts were assessed by qRT-PCR.

(C) 1000 μg PKH67 labeled TDEs were injected into the footpad of wild type C57BL/6 mice and isolated PKH67+ or PKH67- DCs in popliteal lymph nodes (LN). Expression of the indicated transcripts were assessed by qRT-PCR.

(D) BMDCs were incubated with or without TDEs (400 μg/ml) in the presence or absence of GSK3787 (1 μM) for 48 h. Intracellular lipid content was assessed 24 h later via Bodipy 493/503 staining.

(E and F) BMDCs were treated as in (D) with the addition of 2 mg/ml OVA, which were subsequently washed off the antigen and co-cultured with OT I $CD8^+$ T cells for another 3 days. Proliferation of OT I $CD8^+$ T cells was analyzed by CFSE dilution (E). The production of IFN-γ by $CD8^+$ T cells was assessed by ELISA (F).

**P* < 0.05; ****P* < 0.001; *****P* < 0.0001; ns, not significant. (B) and (C) were analyzed with two-way ANOVA. Other data were analyzed with one-way ANOVA. Error bars represent SEM. The error bars of (F) represents SD.

Figure S5

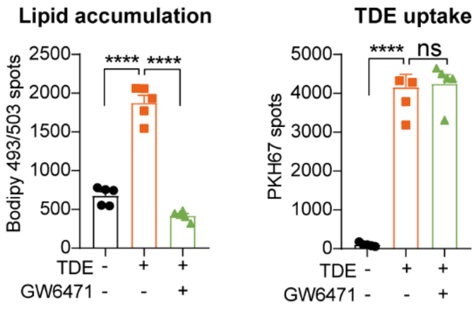

Figure S5 (related to Figure 5). Lipid accumulation and TDE uptake of BMDC

BMDCs were treated with or without TDEs (400 μg/ml) in the presence or absence of GW6471 (15 μM) for 8 h, the intracellular lipid content and TDE uptake were detected by Bodipy493/503 or PKH67, and recorded by Opera Phenix™ High Content Screening System. Each dot represents a biological repeat.

****$P < 0.0001$; ns, not significant. Data were analyzed with one-way ANOVA. Error bars represent SEM.

Figure S6

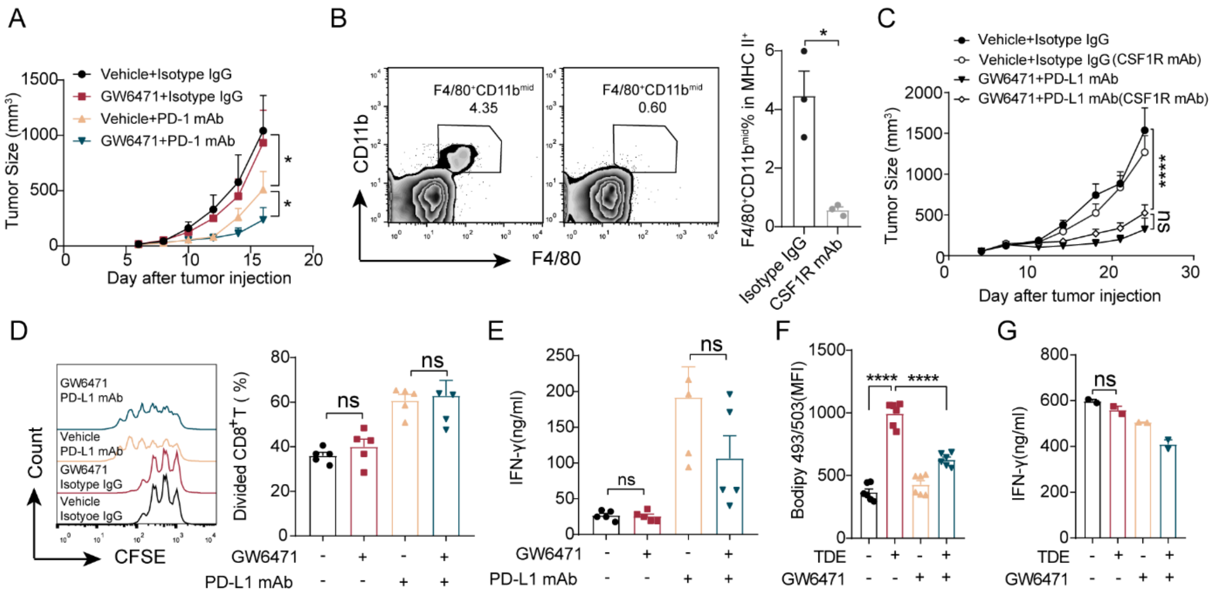

Figure S6 (related to Figure 1 and 6). Macrophages are dispensable for the combination therapy

(A) B16/F10 bearing C57BL/6 mice were treated with PD-1 mAb (200 µg/dose per mouse on day 3,6,9) and GW6471 (10 mg/kg, from day 6, once every other day). Tumor growth curve was shown (*n*=5 per group).

(B) MC38-OT I bearing C57BL/6 mice were treated with PD-L1 mAb (200 µg/dose per mouse on day 7,10,17) and GW6471 (10 mg/kg, from day 11, once every other day). CSF1R mAb 200 ug/mice was injected intraperitoneally on day 7, 10 and 17. 24 h after the third CSF1R mAb injection, $CD45^+$MHC $II^+CD11b^{mid}F4/80^+$ cells were measured by flow cytometry. Each dot represents an individual mouse.

(C) Tumor growth curve was shown (*n*=5 per group).

(D and E) The isolated TAM ($CD45^+$MHC $II^+F4/80^+$) were treated with 2 mg/ml OVA overnight, then co-cultured with OT I $CD8^+$ T cells for 3 days. Proliferation of $CD8^+$ T was analyzed by CFSE dilution (D) and the production of IFN-γ of $CD8^+$ T cells was analyzed by ELISA (E).

(F) BMDMs were differentiated by M-CSF for 7 days, and lipid level in BMDMs treated with TDEs (400 µg/ml) for 24 h in the presence or absence of GW6471.

(G) BMDMs were treated with 2 mg/ml OVA in the presence or absence of TDEs (400 µg/ml) for 48 h, which were subsequently washed off the antigen and co-cultured with OT I $CD8^+$ T cells for 3 days. The production of IFN-γ by $CD8^+$ T cells was analyzed by ELISA.

*$P < 0.05$; ****$P < 0.0001$; ns, not significant. Data were analyzed with 2-tailed *t* test. Error bars represent SEM. The error bar of (G) represents SD.

Table S1

Endocytosis inhibitor	Mechanism	Concentration and incubation time
chlorpromazine	interacts with clathrin	25 uM, 30 min
filipin III	binds to 3' β-hydroxysterol	8 uM, 30 min
wortmannin	blocks PI-3 kinase	100 nM, 60 min
chloroquine	increases endosomal pH	100 uM, 30 min
bafilomycin A	blocks v(H+)ATPase	100 nM, 60 min
nocodazole	depolymerizes microtubules	20 uM, 30 min
N-ethylmaleimide	alkylates sulfhydryl	2 mM, 30 min
FCCP	an uncoupling agent	10 uM, 15 min
NaN_3	depletes cellular ATP	10 mM, 15 min

Table S1 (related to Figure 3). The inhibitors used to block TDEs endocytosis.
